# Plastic Additives in Single-Use and Reusable Menstrual Products: Potential Implications for Human Health and the Environment

**DOI:** 10.1021/acs.est.5c09064

**Published:** 2025-10-15

**Authors:** Lara Cioni, Júlia Calvo, Ethel Eljarrat

**Affiliations:** Environmental and Water Chemistry for Human Health (ONHEALTH), 203229 Institute of Environmental Assessment and Water Research (IDAEA) - CSIC, Barcelona, ES-08034, Spain

**Keywords:** Phthalates, organophosphate esters, alternative plasticizers, consumer products, human exposure, dermal exposure, risk assessment, environmental impact

## Abstract

Menstrual products are essential for half of the world’s population during menstruation, but recent studies have found that these products can contain chemicals of concern for human health. The present study detected three classes of plastic additives, phthalates (PAEs), organophosphate esters (OPEs), and alternative plasticizers (APs) in both single-use (sanitary pads, panty liners, and tampons) and reusable (reusable sanitary pads, menstrual underwear, and menstrual cups) menstrual products. Concentrations were between < LOD-42193 ng/g, < LOD-4068 ng/g, and 95.6–13857 ng/g for PAEs, OPEs, and APs, respectively. EDI calculations showed that dermal contact with some menstrual products might be a significant exposure pathway (0.00–3105 ng/kg bw/day for PAEs; 0.00–237 ng/kg bw/day for OPEs; 0.01–7140 ng/kg bw/day for APs, depending on the product). Additionally, risk assessment calculations showed that using some of these products might pose a risk to human health (cancer risk estimates > 10^–6^). However, these calculations were based on a worst-case scenario, assuming 100% dermal uptake, which might not reflect real-life situations. Environmental impact calculations showed that menstrual products might contribute to the release of plastic additives into the environment once these products enter the waste cycle or are washed to be reused.

## Graphical abstract

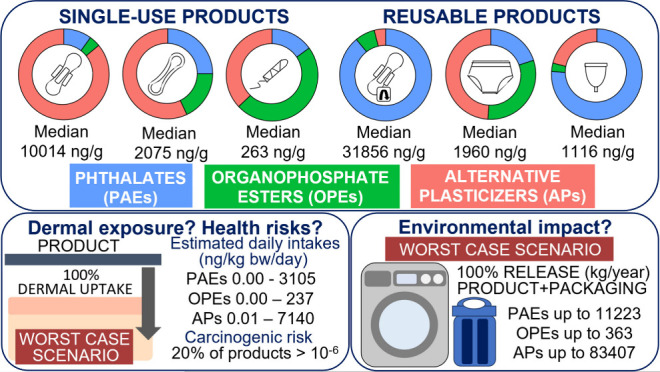

## 1 Introduction

Menstrual products are essential for half of the world′s population to maintain hygiene, prevent infections, provide comfort, and allow access to educational, occupational, and social activities during menstruation.^1^ However, menstrual products can contain chemicals of concern for human health, such as dioxins, pesticides, per- and polyfluoroalkyl substances, and phthalic acid esters (PAEs).^2–8^ From a human exposure perspective this is a concern, since these products are used for several days each month from menarche (average age 12 ^9,10^ ) to menopause (average age 51 ^11^), and the vaginal and vulvar tissues have a higher chemical absorption capacity compared to other skin tissues.^12^ Additionally, these products are used during fertile life stages, which can be a sensitive time frame for human exposure, since exposure to endocrine-disrupting chemicals (EDCs) can be relevant for gynecological and reproductive conditions, such as endometriosis, adenomyosis, and uterine fibroids.^13,14^

Among the chemicals of concern found in menstrual products there are PAEs,^3–5,7^ a group of plastic additives including chemicals classified as EDCs.^15,16^ Exposure to some PAEs, like the bis­(2-ethylhexyl) phthalate (DEHP), has also been associated with increasing risk of cancer.^16^ Due to these concerns, some PAEs have been regulated in several countries.^17^ In particular, since 2020, the European Union limited the use of 4 PAEs, including DEHP, dibutyl phthalate (DnBP), diisobutyl phthalate (DiBP), and benzyl butyl phthalate (BBzP), in consumer products,^18,19^ to concentrations below 0.1% by weight in plasticized materials.^20^ These regulations have been shown to be effective in reducing human exposure to these chemicals.^21–25^ However, PAEs are still widely used in products, and high molecular weight PAEs, like the diisononyl phthalate (DiNP) and the diisodecyl phthalate (DiDP), have emerged as alternatives to the regulated ones, even if these substances are also showing associations with potential adverse effects.^26^

Despite the detection of high concentrations of PAEs in menstrual products, data on the occurrence of other plastic additives are lacking. Among plastic additives, two additional classes of interest for human exposure are organophosphate esters (OPEs) and alternative plasticizers (APs). These two classes of plastic additives have been previously detected in consumer products (face-masks,^27^ textiles,^28^ and food contact materials^29^) but have not been analyzed in menstrual products, so far. The presence of OPEs in consumer products is a concern because these compounds have been linked to various health effects, including immunotoxicity, neurotoxicity, and endocrine disruption.^30^ Additionally, chlorinated OPEs, like tris­(2-chloroethyl) phosphate (TCEP) and tris­(2-chloroisopropyl) phosphate (TCIPP), have been classified as carcinogenic.^30^ APs include a variety of novel plasticizers, such as citrates, adipates, and trimellitates, which have become widely used as a response to the toxicological concerns surrounding OPEs and PAEs.^31,32^ However, information about APs toxicological properties is still scarce, and recent studies are showing that some of these chemicals can also be linked to adverse effects. For example, acetyl tributyl citrate (ATBC) and tri-*n*-butyl citrate (TBC) showed neurotoxic effects in animal studies and ATBC, diisononyl cyclohexane-1,2-dicarboxylate (DINCH), and di­(2-ethylhexyl) (DEHA) have shown potential thyroid disruption.^33,34^

The goals of the present study were (1) to investigate the occurrence of 3 classes of plastic additives, including PAEs and, for the first time, OPEs and APs in different types of single-use and reusable menstrual products; (2) to assess the contribution of dermal contact with these products to plastic additives human exposure; (3) to evaluate the human health and environmental impacts associated with the use of different menstrual products.

## 2 Materials and Methods

Chemicals and consumables are listed in Supporting Information.

### 2.1 Sample Selection

A total of 41 menstrual products purchased in 2024 were analyzed. Most products were purchased from local supermarkets (Barcelona, Spain) and online stores with national distribution, ensuring that the sampling was representative of the menstrual products market in Spain. Most brands sampled are also distributed in EU countries other than Spain, and brands with an online store provide distribution to other countries within and outside the EU. Some samples were obtained from products distributed for free to residents of the Catalan region (Spain) as part of a regional government health initiative to promote access to reusable menstrual products.^35^ Since these products were obtained from brands distributed in Spain, these are also considered representative of the Spanish market. The products selected included single-use (10 sanitary pads, 8 panty liners, and 9 tampons) and reusable (4 reusable sanitary pads, 4 menstrual underwear, and 6 menstrual cups) products. This distribution reflects product usage patterns in Spain, where sanitary pads are the most used (60.6%), followed by panty liners (49.7%), menstrual cups (48.4%), tampons (42.6%), reusable pads (15.0%), and menstrual underwear (8.7%).^36^ Additionally, to ensure the sampling was representative of the menstrual products market, samples for each product type were selected to cover different brands, product lines (products from the same brand, marketed with different names because of different properties, like scent and comfort), sizes, and prices (detailed information in Table S2). Brands included were both commercial and private brands from different supermarket chains, allowing the coverage of a wide range of price categories. Including products with different costs (including some distributed for free) supports representativeness of the sampling, since product cost significantly influences menstrual product choice due to the widespread problem of menstrual poverty.^36^ For the single-use products, the plastic packaging was also analyzed. Single-use products and packaging were analyzed separately.

### 2.2 Analytical Methodology

For analysis, representative portions of each menstrual product were cut into pieces and weighed in glass tubes to reach a sample weight of 0.1 g. For sanitary pads, panty liners, reusable sanitary pads, and menstrual panties, 1 cm^2^ squares were cut from different parts of the products (Figure S1-a), always including all layers (layer in contact with the skin, absorbent layer, and external layers, which included adhesives in single-use products) to obtain concentrations representative of the whole product. Tampons, menstrual cups, and packaging samples were cut in small pieces of approximately 1 cm^3^ (tampons and cups) or 1 cm^2^ (packaging), and pieces were randomly selected to achieve the sample amount needed (Figure S1-b).

The extraction for menstrual products and packaging was adapted from a method for plastic additives in face masks.^27^ Briefly, samples were spiked with 15 μL of 1 ng/μL plastic additives internal standard mixture (Table S1), left to equilibrate for at least 2 h, and extracted twice with 40 mL of hexane:acetone (1:1) using sonication for 15 min. Extracts were filtered with a glass funnel filled with glass wool to remove large fibers, combined, and evaporated with a Turbovap evaporator to reach a volume of ∼5 mL. The extracts were transferred to 2 mL vials with Pasteur pipettes in multiple steps, in which the solvent was gradually evaporated with nitrogen to allow the transfer of the entire extract. The empty extract tubes were rinsed with ∼3 mL of clean hexane:acetone to ensure quantitative transfer. The samples were then evaporated to incipient dryness using a gentle flow of nitrogen, and 500 μL of methanol was added. Samples were filtered with a PTFE 0.2 μm syringe filter and stored at −20 °C until analysis. Plastic additives were analyzed using an ultrahigh pressure liquid chromatography triple-quadrupole mass-spectrometer (UHPLC-MS/MS) with a previously published method^37^ (more details in Supporting Information).

### 2.3 Analytical Method QA/QC

To minimize blank contamination, the use of plastic labware was avoided using glassware washed with acetone and ethanol and burnt at 400 °C for 4 h. Since contamination from plastic additives cannot be completely avoided, each batch of samples included a method blank (empty extraction tube). Limits of detection (LODs) were established as the minimum analyte quantity that produced a signal-to-noise ratio of 3. For samples above the LOD, the blank concentrations were subtracted. The method was validated in terms of recovery, sensitivity, and reproducibility (see SI). Recoveries were between 53 and 94% for PAEs, 44–83% for OPEs, and 47–105% for APs (Table S3). For some analytes (TEP, TPrP, RDP, 4IPPDPPP, TECP, ATEC, DIPA), recoveries between 40 and 50% were considered acceptable, since reliable quantification was ensured using a matching internal standard or a close eluting internal standard for quantification (Table S1). LODs were between 0.72 and 71.9 ng/g for PAEs, 0.06–12.5 ng/g for OPEs, and 0.83–93.4 ng/g for APs (Table S4). The RSDs for the triplicate recovery experiments were <20% for all analytes except DBP, for which a higher variability can be expected since this compound is quantified as the sum of two isomers (DiBP and DnBP) (Table S3). In addition, for sanitary pads, which present a heterogeneous composition, reproducibility of the method within the same product and within the same batch was evaluated. The reproducibility within the same product was <15% and within the same batch was <25% (Table S5), showing that the sampling strategy was representative and that no variability in plastic additives content was to be expected within a product batch.

### 2.4 Dermal Exposure Calculations and Human Health Risk Assessment

The concentrations of plastic additives found in menstrual products were used to calculate the estimated daily intakes (EDIs) through dermal contact with these products (i.e., the intake of plastic additives during 1 single day of product use), using eq 1, adapted from previous studies.^3,4,6^

1 $$EDI\;\left(\frac{ng}{kg\;bw*day}\right)=[C\;\left(\frac{ng}{product}\right)*N\;\left(\frac{product}{day}\right)*\mathrm{ERF}\;\left(\mathrm{dimensionless}\right)*\mathrm{AF}\;\left(\mathrm{dimensionless}\right)]/[NU\left(\dim ensionless\right)*BW\left(kg\;bw\right)]$$

where *C* is the plastic additive concentration in ng/product (obtained multiplying the ng/g concentration by the product weight); *N* is the number of products used in 1 day; ERF is the easily releasable fraction, i.e., the fraction of chemical that is released from a product and reaches the skin; AF is the absorption factor, i.e., the fraction of chemical that from the skin surface can be absorbed and reach systemic circulation; NU is the number of uses for an individual product; and BW is the average body weight of women living in Spain expressed in kg. Table S6 provides the values used for each parameter. Given that the average body weight of women varies from menarche until menopause, EDIs were calculated for 3 different age groups: 12–18 years old, 19–40 years old, and 41–51 years old.^38,39,40^ Since dermal exposure through menstrual products is still poorly understood, plastic additives ERFs for menstrual products and AFs for the vaginal and vulvar tissues are not available in the literature. Therefore, ERFs and AFs were set to 1 for all plastic additives, assuming a worst-case scenario of 100% release of the additives from the menstrual products and 100% absorption through the skin. For some of the additives included in this study, there are published ERFs for clothing^40,41^ and AFs for normal skin.^42,43^ However, while using a worst-case scenario assumption introduces uncertainties, using these ERFs and AFs for menstrual products was considered inappropriate. ERFs for clothing are unreliable for textile-based menstrual products, which consist of multiple layers, unlike single-layer garments. Moreover, for products like sanitary pads, panty liners, tampons, and menstrual cups, ERFs likely differ due to different material compositions. Similarly, using AFs for regular skin would underestimate exposure, as vaginal and vulvar skin show higher absorption, particularly for low molecular weight compounds.^12^ Under this worst-case scenario assumption, the introduction of the number of products used in a day (*N*) in eq 1 is equivalent to assuming 100% release from each individual product. Zeng et al.^40^ observed ERFs between 0.39 and 0.97 from t-shirts in dermal migration experiments with a contact time of 10 h, and Wang et al.^41^ observed ERFs between 0.06 and 0.75 in dermal migration experiments with a duration of 8 h. Therefore, it is possible that for some chemicals in menstrual products the ERFs could be close to 100% during the time that only one product is used (this time ranges from 4 to 6 h). The NU variable was added to the EDI denominator to account for the fact that each reusable product will release 100% of its content of plastic additives over its entire product lifespan rather than in a single day of use. NU is a dimensionless parameter equal to 1 for single-use products, while for reusable products NU was estimated by multiplying the average product lifespan (5 years for reusable sanitary pads and menstrual underwear, 10 years for menstrual cups^44^) by the average number of menstrual bleeding days in 1 year (50.3 days).

For compounds with established toxicological thresholds, risk assessment was performed in terms of noncarcinogenic and carcinogenic effects using established guidelines.^45–47^ Briefly, noncarcinogenic risk was assessed by calculating a hazard quotient (HQ) for each plastic additive. The HQ was calculated by dividing the average daily dose (ADD) by the noncancer health reference dose (RfD), minimal risk level (MRL), or tolerable daily intake (TDI) (Table S7). For those compounds with more than one toxicological threshold defined, the most conservative value was chosen. A potential noncarcinogenic risk is considered when the HQ is higher than 1; otherwise, the risk is considered negligible.

Since the RfDs, MRLs, and TDIs used are derived from chronic exposures, the ADD had to be calculated for a chronic exposure duration (1 year or longer). The ADD was calculated with eq 2 using established guidelines:^48^

2 $$ADD\;\left(\frac{ng}{kg\;bw*day}\right)=\frac{EDI\;\left(\frac{ng}{{kg\;bw}^{*}day}\right)*\mathrm{EF}\;\left(\frac{day}{year}\right)*\mathrm{ED}\;\left(\mathrm{year}\right)}{AT\;\left(day\right)}$$

where EDI is the estimated daily intake (eq 1), EF is the exposure frequency, ED is the exposure duration, and AT is the averaging time. As mentioned above, EDI is the intake of plastic additives during 1 single day of product use. EF is the number of days these products are used in a year. EF was set to 365 for panty liners (these products can be used daily), while for all other products (only used during menstruation), the average menstrual bleeding duration (50.3 days/year) was used. ED is the time that an individual is exposed to plastic additives through the use of menstrual products. As for EDIs, ADD calculations were age-specific due to changes in body weight between menarche and menopause, and ED was set to the exposure years considered: 7 years (12–18 years old), 22 years (19–40 years old), and 11 years (41–51 years old). AT is the time over which exposure is averaged and for noncarcinogenic risk is equal to the ED.^48^ Therefore, AT was set to 2190 days (12–18 years old), 8030 days (19–40 years old), and 4015 days (41–51 years old).

Carcinogenic risk was calculated only for carcinogenic additives with an available oral slope factor (SFO) (Table S7). Since the SFO represents the incremental cancer risk over a lifetime,^49^ carcinogenic risk was evaluated by multiplying the lifetime average daily dose (LADD) of a plastic additive by the specific SFO and by 10^–6^ for unit conversion. If the product is lower than 10^–6^, the cancer risk is considered negligible; if it is between 10^–6^ and 10^–4^, there is a potential cancer risk; and if it is higher than 10^–4^ there is a high-potential cancer risk. The LADD was calculated using eq 3:

3 $$LADD\;\left(\frac{ng}{kg\;bw*day}\right)=\frac{\sum \left({EDI}_{age\;i}\;\left(\frac{ng}{kg\;bw*day}\right)*{EF}_{age\;i}\;\left(\frac{day}{year}\right)*{ED}_{age\;i}\;\left(\mathrm{year}\right)\right)}{AT\;\left(day\right)}$$

where EDI is the estimated daily intake (eq 1), EF is the exposure frequency, ED is the exposure duration, and AT is the averaging time. For carcinogenic risk, a cumulative dose over a lifetime is considered. Therefore, exposure at different life stages of a menstruator is summed together, and AT is set to a lifetime (as established by US EPA guidelines^48^), using the average life expectancy of women in Spain in 2024 (86.4 years).^50^

For all chemicals included in this study, the toxicological thresholds are defined for ingestion and not for dermal uptake since there is not sufficient data from human and animal studies focusing on this exposure pathway. Therefore, the present risk assessment has uncertainties related to extrapolation from oral to dermal exposure.

### 2.5 Environmental Impact Assessment

The environmental impact was assessed by calculating the plastic additives emissions from menstrual products used in Spain using eq 4:

4 $$Additives\;emissions\;\left(\frac{kg}{year}\right)=[\left(C\;\left(\frac{ng}{product}\right)+C_{P}\;\left(\frac{ng}{product}\right)\right)*N\;\left(\frac{product}{day}\right)*UF\;\left(\frac{days}{year}\right)*NW*10^{-12}]/[NU]$$

where *C* is the plastic additive concentration in ng/product; *C*_P_ is the plastic additive concentration in the single-use products packaging in ng/product; *N* is the number of products used in 1 day (Table S6); UF is the number of days in a year in which the products are used (365 days for panty liners, 50.3 days for the other products only used during menstruation); NW is the number of women in Spain that menstruate (12,154,865 women with age between 12, average age of menarche, and 51 years, average age of menopause, in 2024 ^51^); 10^–12^ is the conversion factor from ng to kg; NU is the number of uses for an individual product (Table S6).

### 2.6 Statistical Analyses

Statistical analyses were performed using R version 4.3.2 (R Core Team). Prior to statistics calculations, concentrations below LOD were substituted with LOD/√2. Differences in concentrations of ∑PAEs, ∑OPEs, ∑APs, and total plastic additives between different types of menstrual products were assessed using the Kruskal–Wallis rank sum test and pairwise comparisons with the Wilcoxon rank sum exact test with correction for multiple testing. Associations between plastic additives concentrations in the menstrual products and in the packaging were evaluated using Spearman’s rank correlation coefficients only for those compounds with a detection frequency ≥ 50% in both products and packaging (TNBP, ATBC, and TBC). Statistical significance was set at *p* < 0.05.

## 3 Results and Discussion

### 3.1 Plastic Additives Occurrence

All menstrual products had detectable concentrations of plastic additives, and a total of 5 PAEs, 16 OPEs, and 7 APs were detected (Tables S8–S10). PAEs were detected in all reusable sanitary pads and menstrual cups, but showed lower detection frequencies in other products. OPEs were detected in 100% of all products, except menstrual cups (detection frequency: 17%). Finally, for APs the detection frequency was 100% across all products, reflecting a more widespread use. Indeed, many APs are used as substitutes for PAEs and OPEs that are regulated or considered of concern for environmental and human health.^31,32^

Plastic additives concentrations varied depending on the product type (Figure 1). Differences were observed in terms of both ng/g and ng/product concentrations (obtained by multiplying ng/g concentrations by the product weights) (Figure S2). For total plastic additives, significant differences were observed among different products (Kruskal–Wallis rank sum test: *p*-value < 0.05, Table S11). The highest total plastic additive concentrations were found in reusable sanitary pads (median: 31856 ng/g; range: 6140 −47174 ng/g) followed by sanitary pads (median: 10014 ng/g; range: 4310–16197 ng/g) ≈ panty liners (median: 2075 ng/g; range: 271–13998 ng/g) ≈ menstrual underwear (median: 1960 ng/g; range: 424–4283 ng/g) ≈ menstrual cups (median: 1116 ng/g; range: 326–2454 ng/g) > tampons (median: 263 ng/g; range: 243–1027 ng/g). (Figure 1, Table S11).

**Figure 1:**
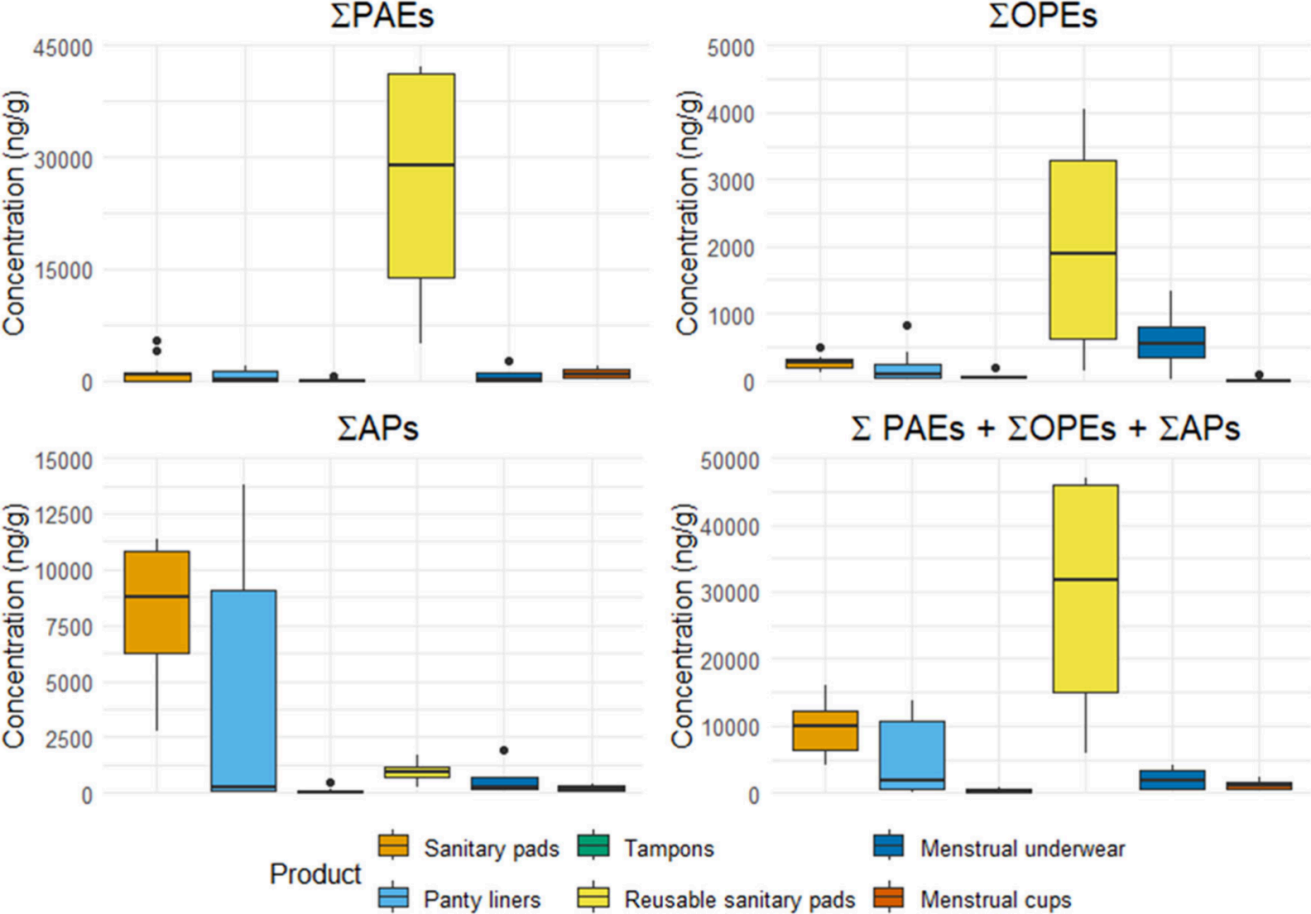
∑ PAEs, ∑ OPEs, ∑APs, and total plastic additives concentrations (∑ PAEs + ∑ OPEs + ∑ APs) (ng/g) in sanitary pads, panty liners, tampons, reusable sanitary pads, menstrual underwear, and menstrual cups (note the different scales).

Considering the different classes of additives analyzed, reusable sanitary pads had the highest concentrations of PAEs (median: 28856 ng/g; range: 5019–42193 ng/g) and OPEs (median: 1906 ng/g; range: 158–4068 ng/g), but the highest concentrations of APs were observed in the single-use sanitary pads (median: 8873 ng/g; range: 2830–11455 ng/g). Tampons had the lowest concentrations of PAEs (median: < LOD ng/g; range: < LOD-616 ng/g) and APs (median: 145 ng/g; range: 113–525 ng/g), while the lowest OPEs concentrations were found in menstrual cups (median: < LOD ng/g; range: < LOD-98.0 ng/g) (Figure 1). The differences in concentrations between different products were significant for all classes of additives (Kruskal–Wallis rank sum test: *p*-value <0.05). However, even if clear differences in median concentrations were observed among product types, pairwise comparisons showed statistically significant differences only between certain types (Tables S12, S13, S14). The differences in concentrations might be attributed to product design. Tampons consist of an absorbent material covered by a thin synthetic fiber to facilitate application, while sanitary pads and panty liners have multilayer compositions with one or more plastic layers. Despite the similar design of sanitary pads and panty liners, their composition can differ, since sanitary pads are designed for regular/abundant menstrual flow, while panty liners are made to retain small losses of blood/urine. Reusable sanitary pads and menstrual underwear are different from single-use products since these are made of textiles, often including synthetic fibers and a waterproof layer. Lastly, menstrual cups differ from all other products and are made solely of silicone or thermoplastic elastomer (TPE). Differences in concentrations might also be due to the use of different polymers and materials. However, since most products are composed of a combination of multiple polymers that varies between different brands (Table S2), it is not possible to conclude if differences in plastic additive concentrations are driven by the materials used.

APs were the main plastic additives in sanitary pads, panty liners, and menstrual underwear, but not in tampons, in which OPEs were the dominant compounds, and reusable sanitary pads and menstrual cups, in which PAEs were the dominant compounds (Figure 2). As mentioned earlier, APs are used as replacements of PAEs and OPEs in many applications, including plastic and textile materials,^40,52^ and their more widespread detection might reflect this shift. In most menstrual products, PAEs concentrations were higher than OPEs, similar to other plastic-based products, such as face masks,^27,53^ textiles,^17,54^ and food contact materials.^29,55^ Only in tampons and menstrual underwear were OPEs found in higher concentrations than PAEs. Despite the regulation of some PAEs, these compounds are still widely used in consumer products. It has been hypothesized that the presence of PAEs in menstrual products, such as sanitary pads and panty liners, might be coming from the plastic materials used on the top/bottom layers. PAEs might also be used in sanitary pads and panty liners in the adhesives added to these products or as fragrance fixatives, since previous studies have observed higher PAEs concentrations in sanitary pads with a scent applied compared to those without a scent.^4,5^ The sample selection of the present study included both products with and without a scent applied, but no clear differences in PAEs concentrations and profiles were observed between scented and unscented products (Figure S3). However, since the presence of scents was not a factor driving the sample selection, this comparison might be limited by the low number of samples of sanitary pads without a scent and panty liners with a scent applied. PAEs are also widely used in the textile industry to produce synthetic fibers and to give textiles waterproof properties.^17^ A waterproof layer is always included in reusable menstrual products, and most of the products included in this study had at least one textile layer made of a synthetic fiber, such as rayon, polyester, and elastane (Table S2). Additionally, PAEs’ presence in menstrual underwear and reusable sanitary pads might be due to the presence of these compounds in dyes, textiles inks, and other processing aids and water used during textiles and product production.^17^ Previous studies also hypothesized that PAEs in menstrual products might be coming from the product packaging. In the present study PAEs, OPEs, and APs were detected in the packaging of single-use products (Table S15), and a positive correlation between the concentrations in the product and in the packaging was significant only for TBC and ATBC in panty liners (Table S16). This suggests that the packaging might indeed be a source of plastic additives in menstrual products but that this might depend on the materials used in the product and/or packaging since associations were only observed for panty liners.

**Figure 2:**
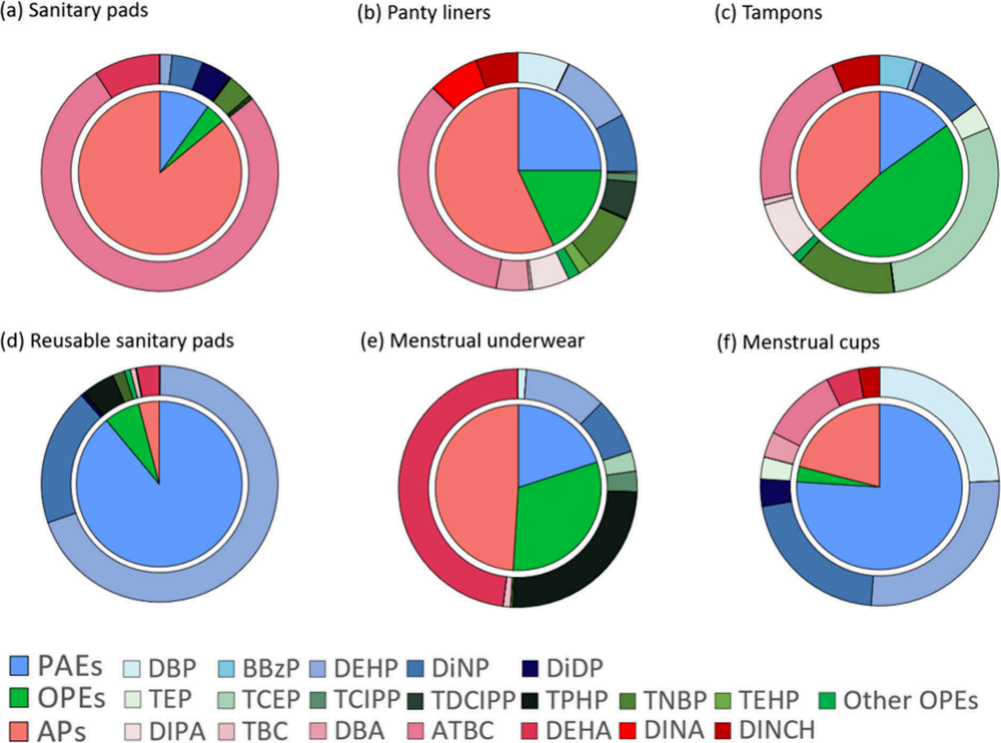
Average percentage contribution of PAEs, OPEs, and APs to total plastic additives concentrations in sanitary pads, panty liners, tampons, reusable sanitary pads, menstrual underwear, and menstrual cups.

Variability in composition between different products was also observed within the three classes of additives analyzed. Among the 5 PAEs detected in menstrual products, DEHP and DiNP were the major components in reusable sanitary pads and menstrual underwear (Figure 2). DEHP concentrations in reusable sanitary pads (median: 22825 ng/g; range: 4913–41929 ng/g) were at least 1 order of magnitude higher than in menstrual underwear (median: 161 ng/g; < LOD-400 ng/g). DiNP was only detected in one sample of reusable sanitary pads (14135 ng/g) and one sample of menstrual underwear (2077 ng/g) from the same brand, but at high concentrations. As for DEHP, DiNP concentrations in reusable sanitary pads were higher than in menstrual underwear. This is consistent with several studies reporting DEHP and DiNP among the main PAEs detected in textile materials.^56–59^ DEHP is the PAE consumed in greatest quantities by the textile industry, and, similar to our findings, most of the literature on textile-based products found DEHP to be the PAE present in the highest concentrations.^17^ An additional PAE, DiDP, was detected in all reusable sanitary pads but at low concentrations (median: 91.1 ng/g; 54.7–192 ng/g) compared to DEHP and DINP. This PAE has also been detected in other textile products.^57,59,60^ DEHP (median: 116 ng/g; 36.1–1003 ng/g) and DiNP (median: < LOD ng/g; < LOD-1477 ng/g) were also major components in menstrual cups, but with lower concentrations than other reusable products. Additionally, in menstrual cups DBP was found as a major additive, since it was detected in 5 out of 6 menstrual cups (median: 138 ng/g). DiDP was also detected in half of the menstrual cups. Interestingly, DiNP was detected only in TPE cups (Figure S4). The presence of PAEs in menstrual cups can be expected since these compounds are often used to improve the flexibility of plastic materials. For single-use products, DBP, DEHP, DiNP, and DiDP were also the compounds most frequently detected, but the detection frequencies were lower, and their contribution changed depending on the product type.

However, PAEs results in sanitary pads, panty liners, and tampons differed from those of previous studies^3–5^ (Tables S17, S18). DBP was not detected in single-use sanitary pads from our study but was detected in all sanitary pads analyzed in previous studies. These studies reported the DBP isomers separately with median concentrations between 73.0 and 1424 ng/g for DiBP and between 83.3 and 909 ng/g for DnBP. Additionally, DEHP (detected only in one sample from our study) and DMP (not analyzed in our study) were also detected in most of the sanitary pads from previous studies. PAEs in tampons and panty liners were only reported in one study by Gao and Kannan.^3^ For DEHP, concentrations from the Gao and Kannan study (mean: 744 ng/g for tampons, 2070 ng/g for panty liners) were at least 1 order of magnitude higher than those in our study (mean: 14.5 ng/g for tampons, 121 ng/g for panty liners). In the Gao and Kannan study, DiBP and DnBP were quantified separately and found to be in both product types. In our study, DiBP and DnBP were not found in tampons but were quantified together in several panty liners with concentrations lower than those reported in the literature. These differences, observed between our study and previous literature reports, might be due to changes in PAEs legislation and production since the products in our study were collected during 2024, while the products in previous studies were bought between 2016 and 2019.^3–5^ The differences might also be due to differences in PAEs legislation between the countries where the samples were purchased. For example, the EU limits the application of BBzP, DBP, DEHP, and DiDP in most consumer products, while the US regulates the same PAEs only in child toys.^61^ Further, the analyzed PAEs in the current study differ from those of previous studies, and this discrepancy might also affect the differences observed in terms of ∑ PAE concentrations.

OPEs also differed between different menstrual products (Figure 2). TNBP was detected only in single-use products and was the dominant OPE in sanitary pads and panty liners with concentrations between 110 and 319 ng/g (median: 236 ng/g) and between < LOD-193 ng/g (median: 22.4 ng/g), respectively. TNBP was also widely detected in tampons (detection frequency: 78%) but at lower concentrations (median: 11.1 ng/g; range: < LOD-99.7 ng/g). A wide variety of other OPEs were detected in sanitary pads and panty liners, but with detection frequencies <50% and concentrations at least 1 order of magnitude lower than TNBP (Table S9). TNBP is one of the OPEs most widely used as plasticizer and this might explain its wide detection only in single-use products.^62^ In tampons, the dominant OPE was TCEP (median: 24.6 ng/g; range: 11.7–82.7 ng/g), which was detected in all samples with concentrations comparable to those of TNBP. TCEP was also detected in one sample of menstrual underwear at a high concentration (216 ng/g). However, the main OPE in reusable sanitary pads and menstrual underwear was TPHP, which was detected in 100% of both types of products at high concentrations (median: 820 ng/g in reusable sanitary pads; 316 ng/g in menstrual underwear). TPHP is known to have applications in textiles and textile coatings.^62^ In menstrual cups, TEP was the only OPE detected, but only in one of the samples analyzed.

Lastly, among the APs, ATBC was the dominant compound in all single-use products and menstrual cups (Figure 2). The highest ATBC concentrations were found in sanitary pads (range: 2714–11314 ng/g) and panty liners (range: < LOD-13563). ATBC is a popular alternative to DEHP and is currently widely used as plasticizer in various applications, including medical devices, cosmetics, and food packaging.^63^ Therefore, its widespread detection at high concentrations in plastic-based menstrual products is perhaps not surprising. In textile-based menstrual products, ATBC was detected only in one reusable sanitary pad, and the dominant AP was DEHA, which was detected in all samples of these products. DEHA concentrations ranged between 71.3 and 1663 ng/g in reusable sanitary pads and 148–1926 ng/g in menstrual underwear. DEHA was also detected in some samples of sanitary pads and menstrual cups but at lower concentrations (Table S10). DEHA is another popular AP with various applications, including textile materials.^64^ DINCH, detected in a few single-use products, was detected in all TPE menstrual cups and not in the silicone ones (Figure S4). DINCH is a plasticizer used to produce flexible plastic articles,^65^ and this might explain its presence in TPE cups, which need to be flexible to ensure functionality.

### 3.2 Contribution to Human Exposure

To estimate the contribution of dermal contact with menstrual products to plastic additive exposure, the EDIs for the different product types were calculated (Table 1).

**Table 1:** EDIs (ng/kg bw/day) for Dermal Contact with Different Menstrual Products

		age 12–18 years old	age 19–40 years old	age 41–51 years old
product		mean (range)	mean (range)	mean (range)
Sanitary pads	∑PAEs	685 (0.00–3105)	547 (0.00–2478)	502 (0.00–2275)
	∑OPEs	142 (57.3–237)	114 (45.7–188)	104 (42.0–173)
	∑APs	4372 (1322–7140)	3489 (1055–5699)	3204 (969–5232)
Panty liners	∑PAEs	73.4 (0.00–221)	58.6 (0.00–176)	53.8 (0.00–162)
	∑OPEs	37.0 (3.54–141)	29.5 (2.83–113)	27.1 (2.59–104)
	∑APs	604 (4.58–1959)	482 (3.65–1564)	442 (3.35–1436)
Tampons	∑PAEs	31.1 (0.00–142)	24.8 (0.00–113)	22.8 (0.00–104)
	∑OPEs	15.9 (5.19–60.9)	12.7 (4.14–48.6)	11.6 (3.80–44.6)
	∑APs	31.4 (0.81–147)	25.1 (0.65–117)	23.0 (0.60–108)
Reusable sanitary pads	∑PAEs	205 (36.3–359)	164 (29.0–286)	150 (26.6–263)
	∑OPEs	15.8 (0.99–30.0)	12.6 (0.79–24.0)	11.6 (0.72–22.0)
	∑APs	7.49 (2.07–14.6)	5.98 (1.64–11.6)	5.49 (1.52–10.7)
Menstrual underwear	∑PAEs	11.0 (0.00–36.4)	8.78 (0.00–29.9)	8.06 (0.00–26.7)
	∑OPEs	9.46 (0.12–17.8)	7.55 (0.09–14.2)	6.93 (0.08–13.1)
	∑APs	11.5 (2.00–36.4)	9.16 (1.59–29.0)	8.41 (1.46–26.7)
Menstrual cups	∑PAEs	0.44 (0.05–0.90)	0.35 (0.04–0.71)	0.32 (0.04–0.66)
	∑OPEs	0.01 (0.00–0.04)	0.01 (0.00–0.03)	0.01 (0.00–0.03)
	∑APs	0.10 (0.01–0.18)	0.08 (0.01–0.14)	0.07 (0.01–0.13)

The highest EDIs were observed for the youngest age group since the average body weight is the lowest for this group. Looking at the different types of products, the highest EDIs were observed for single-use sanitary pads, and the lowest were observed for menstrual cups for all classes of additives. While some reusable products had higher PAEs and OPEs concentrations than single-use products (Figure 1), the EDIs for these additives in reusable products were lower than in single-use products. This is due to the different use habits. An individual who menstruates will use approximately 6 single-use sanitary pads during a day, and in this study, the worst-case scenario (100% of the additive in the product is released to the skin) was assumed. For reusable products, the worst-case scenario assumption was similar, but it was considered that each product will release 100% of the additives through its entire life cycle. This was achieved by introducing in the EDI formula denominator (eq 1) the number of uses for an individual product and therefore assuming that the plastic additives in reusable products will be released in a constant amount at each use. This is an assumption that might not reflect real-life situations since part of the chemicals will be released during the cleaning of these products between uses. Additionally, the release of plastic additives might change at different stages of use of the products. It has been shown that the highest amounts of microfibers are released from clothes during the first 1–4 washes.^66,67^ This might also be the case for plastic additives. Additionally, the abrasion of reusable menstrual product fibers during washing might also influence the release of these chemicals from the product to the skin.

When dermal contact with menstrual products was compared to other exposure routes, it was observed that the use of some menstrual products might contribute significantly to human exposure to plastic additives. Starting from PAEs, mean EDIs for sanitary pads (Table 1) were comparable to those for exposure through the diet, which is considered the main route of exposure to these compounds.^68–71^ Mean EDIs for dietary intake vary between 104 and 13000 ng/kg bw/day for DEHP^69,70,72–74^ and 212–61000 ng/kg bw/day for DiNP.^69,73,74^ Mean EDIs for reusable sanitary pads were comparable to the lowest estimates reported in the literature for dust ingestion, another major PAEs exposure route (range: 99 and 3980 ng/kg bw/day^75–77^ ). EDIs for PAEs in panty liners, tampons, and menstrual underwear were of the same order of magnitude of the lowest estimates for air inhalation (range: 6.35–360 ng/kg bw/day^75,76,78,79^ ) and dermal exposure measured with skin wipes (range:10–1220 ng/kg bw/day^77,80,81^ ). Only for menstrual cups were ∑ PAEs EDIs well below estimates for other exposure routes. Considering the OPEs, the highest mean EDIs were observed for sanitary pads. The mean EDI for ∑ OPEs in sanitary pads were higher than those reported for dietary intake (range: 0.97–103 ng/kg bw/day^71,82–88^ ), which is considered the main exposure route for OPEs. EDIs for all other types of products except menstrual cups were comparable to those reported for ∑ OPEs through other exposure routes, including air inhalation (range: 1.75–9.2 ng/kg bw/day^89–92^ ), dust ingestion (range: 0.07–23 ng/kg bw/day^89,92,93^ ), and dermal contact with dust (range: 5.89–17 ng/kg bw/day^89,92^ ). For menstrual cups, the EDIs for ∑ OPEs were at least 2 orders of magnitude lower compared to other menstrual products and other exposure routes. Lastly, for APs, comparison with other exposure routes was more difficult to realize due to the limited amount of human exposure data for these compounds. The highest EDIs for APs were observed for sanitary pads, and these might be comparable to EDIs for dietary intake. Two studies, including several APs in different food matrices, estimated median EDIs for ∑ APs through the diet of 244 ng/kg bw/day for adults living in Spain and of 1515 ng/kg bw/day for adults living in Sweden.^71,94^ Additionally, a recent study, analyzing several APs in plant-based food collected in Belgium, Germany and the UK, has calculated a mean EDI for ∑ APs of 610 ng/kg bw/day for a fully vegan diet.^95^ However, other studies on food matrices reporting only few APs found higher EDIs through food consumption (87000 ng/kg bw/day for DINCH intake through the diet^31^ and 30000 ng/kg bw/day for DEHA through soft drink consumption^96^). EDIs for APs through dermal contact with other menstrual products, except menstrual cups, were at least 1 order of magnitude lower than for sanitary pads and were comparable to intake through other APs exposure routes: inhalation of indoor air (15–358 ng/kg bw/day for ATBC; 6.52–12.1 ng/kg bw/day for TBC)^97^ and dust ingestion (2.16–14.4 ng/kg bw/day for ∑APs).^98,99^

In summary, in many cases, the EDIs for plastic additives through dermal contact with menstrual products were comparable to those estimated for other important exposure pathways. However, it is important to highlight that the EDI calculations were performed assuming a worst-case scenario of 100% dermal uptake, which probably differs from a realistic case. Previous studies measuring the release of chemicals from clothing found ERFs ranging between 0.06 and 0.75 for OPEs, 0.28–0.98 for PAEs, and 0.33–0.57 for APs.^40,41^ Similar ERFs values might be expected for menstrual products, especially those made of textiles, but they might vary depending on the material composition. Also, AFs for plastic additives are expected to be lower than 1, since AFs measured these chemicals through regular skin are comprised between 0.13 and 0.75.^42,43^

### 3.3 Human Health Risk Assessment

Noncarcinogenic risk estimates were well below thresholds for toxicological effects for all types of menstrual products (Figure 3). The noncarcinogenic risk was negligible even when the different additives were added together, since the highest HQ value obtained for the total plastic additives was 1.7 × 10^–2^. The noncarcinogenic risk was negligible for all 3 age groups considered, since the risk estimates for the youngest age group (highest EDIs due to the lowest body weight) were well below threshold. On the contrary, for the carcinogenic risk, some products were above the threshold for cancer effects (Figure 3). However, all carcinogenic risk values were below 1 × 10^–4^, above which there would be a high risk. The carcinogenic risk was above threshold for 3 out 10 sanitary pads, 3 out of 8 panty liners, and 2 out of 4 reusable sanitary pads. The cumulative cancer risk was driven by the presence of high concentrations of DEHP and DEHA in these products. It is important to note that this assessment might overestimate the risks for human health since calculations were based on worst-case scenario estimates of 100% dermal uptake. Additionally, this assessment has the drawback that toxicological thresholds used are defined for oral exposure and not for dermal exposure and this adds additional uncertainties. However, it is important to highlight that dermal exposure through menstrual products use is only one of the human exposure pathways to plastic additives. When added to other exposure pathways (e.g., food or dust ingestion), the use of menstrual products might contribute to increasing plastic additives exposure to levels exceeding the thresholds for human health risks for people who menstruate. Further, it is important to consider that these products are used during fertile life stages, and this exposure might be relevant for reproductive health, since exposure to EDCs is a known risk factor for reproductive effects.^13,14^

**Figure 3:**
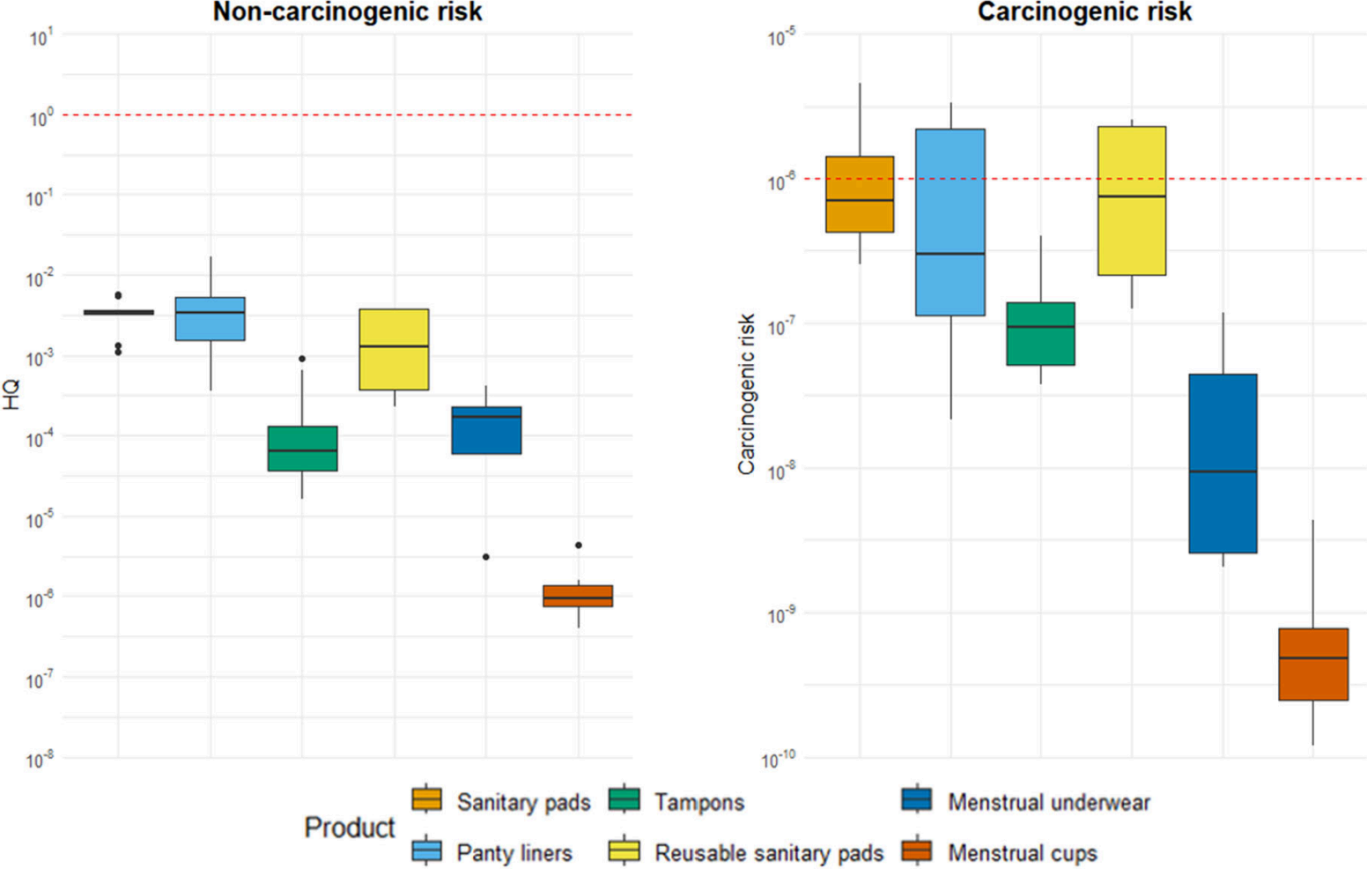
Noncarcinogenic (age group 12–18 years old) and carcinogenic risk estimates for total plastic additives concentrations in sanitary pads, panty liners, tampons, reusable sanitary pads, menstrual underwear, and menstrual cups. The dashed red lines indicate the threshold over which a risk for human health is considered.

### 3.4 Environmental Impact Assessment

Assuming a worst-case scenario of 100% release to the environment, the highest estimates for the release of plastic additives from the use of menstrual products in Spain were found for single-use products (Table S18). The estimates of the release of plastic additives to the environment from sanitary pads (median: 225 kg/year; range 76.9–213127 kg/year), panty liners (median: 82.1 kg/year; range: 4.96–2039 kg/year), and tampons (median: 472 kg/year; range: 12.1–1560 kg/year) were at least 1 order of magnitude higher than for reusable menstrual products. This is due to the higher number of single-use products consumed and to the high concentrations of plastic additives found in the packaging of these products (Table S15). Among the reusable products, reusable sanitary pads (median:7.33 kg/year; range: 1.37–12.5 kg/year) and menstrual underwear (median: 1.05 kg/year; range: 0.12–1.75 kg/year) showed comparable environmental impact. Menstrual cups were the products resulting in the lowest release estimates (median: 0.02 kg/year; range: 0.01–0.03 kg/year). Despite the highest release estimates being found for single-use products, plastic additives release from reusable products might be more concerning (in particular, from reusable sanitary pads, which showed the highest plastic additives concentrations). Single-use products are disposed of as waste directly after use and are expected to enter a landfill or waste incineration. For reusable products, the release of plastic additives to the environment is expected before these products enter the waste cycle, since some chemicals will be released during their washing between uses. Therefore, the plastic additives in reusable products might be released to wastewater and enter the water cycle. This is of concern because wastewater treatment plants are not always efficient in reducing plastic additives contamination.

## 4 Implications

This study detected a wide range of plastic additives, including PAEs, OPEs, and APs, in both single-use and reusable menstrual products. While PAEs have been previously reported in single-use products,^3–5^ this is the first study to detect them in reusable products. Moreover, we report for the first time the presence of OPEs and APs in menstrual products, which had not been investigated until now. Since more than 13.000 plastic additives exist,^100^ it is to be expected that more of these chemicals might be in use in these products. In many menstrual products, APs were the dominant additives, reflecting their widespread use. However, despite their growing use in consumer products, information about human exposure to these chemicals is still scarce, and more information about their toxicological properties is needed.

The EDIs presented in this study show that the dermal contact with menstrual products might be a significant exposure pathway. This is of concern for the health of people who menstruate, as they are already exposed to PAEs, OPEs, and APs through other routes (e.g.; diet, air inhalation). As a consequence, people who menstruate might suffer higher cumulative exposure to these additives, increasing their vulnerability to the associated health effects. However, these calculations were based on a worst-case scenario that probably does not reflect real-life situations. The main factor hindering the calculation of realistic estimates is the lack of knowledge about dermal exposure. To better understand dermal exposure through menstrual products, it is important to test the release of plastic additives from these products under realistic conditions. The release of some of these chemicals from other types of consumer products, such as clothing or other fabric products, has been tested using migration experiments with sweat and sebum to simulate the surface layer of the skin.^41,101^ These migration assays should be adapted to menstrual products to also study the effects of vaginal and menstrual fluids on the release of these chemicals. Additionally, to complete the description of dermal exposure to plastic additives through menstrual products, AFs for vaginal and vulvar tissues should be derived. It has been demonstrated that some plastic additives can be absorbed into the skin. However, the vulvar and vaginal tissues are known to have a higher absorption capacity for chemicals, and new models might be needed to measure absorption through this type of skin. Since the worst-case scenario estimates showed that some products might be associated with carcinogenic risks, future studies focusing on the determination of these dermal exposure parameters are a priority to provide a more realistic risk assessment.

Another important aspect to consider about the presence of plastic additives in menstrual products is the potential environmental impact. The use of all types of menstrual products might contribute to the release of plastic additives to the environment through waste disposal and the washing of reusable products. The highest release of plastic additives from menstrual products was found for single-use products, and this was partly due to their packaging, which is directly introduced in the waste-cycle. Even if the packaging might not contribute significantly to human exposure, since it is directly disposed, strategies to reduce the content of chemicals of concern in the packaging as well as to reduce the packaging amount should be considered to reduce the impact of these products. However, the chemical content is only a part of the environmental impact considerations for these products. This information should complement life cycle assessment studies, considering other environmental aspects to properly assess the impact of menstrual products.

## Supplementary Material



## Notes

Chemicals and consumables; samples information; analytical method QA/QC; exposure parameters and toxicological thresholds for risk assessment; concentrations of individual PAEs, OPEs, and APs in menstrual products and packaging; pairwise comparisons outputs; PAEs concentrations in menstrual products from literature; plastic additives environmental emissions estimates (PDF)
