# Supplementary material for: Plastic Additives in Single-Use and Reusable Menstrual Products: Potential Implications for Human Health and the Environment
Source: Environ Sci Technol. 2025 Oct 15;59(42):22479–91. doi: 10.1021/acs.est.5c09064 (PMC12573791; doi:10.1021/acs.est.5c09064)
Supplement: Supplementary file 1 [file es5c09064_si_001.pdf]

Plastic additives in single-use and reusable menstrual products: potential implications for human health and the environment

Lara Cioni*, Júlia Calvo, Ethel Eljarrat

Environmental and Water Chemistry for Human Health (ONHEALTH), Institute of Environmental Assessment and Water Research (IDAEA) - CSIC, Barcelona, ES-08034, Spain

***Corresponding author**

Lara Cioni - Environmental and Water Chemistry for Human Health (ONHEALTH), Institute of Environmental Assessment and Water Research (IDAEA) - CSIC, Barcelona, ES-08034, Spain

*lara.cioni@idaea.csic.es

Summary: 33 pages, 4 figures, 22 tables

The following information is included: chemicals and consumables (page S4); instrumental analysis (page S9); analytical method QA/QC (page S9, S10).

Figures:

- Figure S1 – Examples of selection of representative portions of menstrual products for analysis: (a) sanitary pads and panty liners; (b) tampons and menstrual cups.
- Figure S2 - ∑PAEs, ∑OPEs, ∑APs and total plastic additives concentrations (∑PAEs + ∑OPEs +∑APs) (ng/product) in sanitary pads, panty liners, tampons, reusable sanitary pads, menstrual underwear and menstrual cups (note the log scale).
- Figure S3 – PAEs concentrations (ng/g) in scented (highlighted in green) and unscented products.
- Figure S4 – Plastic additives concentrations (ng/g) in menstrual cups. Samples from CM-01 to CM-04 are silicone menstrual cups and samples CM-5 and CM-06 are TPE menstrual cups.

Tables:

- Table S1 – Abbreviation, full name, CAS number and manufactures of plastic additives native and labelled standards.
- Table S2 – Detailed information on the menstrual products included in this study (ND=no data). The product line is specified for products from the same brand, marketed with different name because of different properties, like scent and comfort.
- Table S3 – Recovery values (%) for PAEs, OPEs and APs in sanitary pads, menstrual underwear and menstrual cups.
- Table S4 – LODs (ng/g) for PAEs, OPEs and APs in sanitary pads, menstrual underwear and menstrual cups.
- Table S5 – Method reproducibility within the same product and within the same batch for sanitary pads (n=3). Reproducibility can be reported only for compounds detected in the samples analysed.
- Table S6 – Exposure parameters values used for EDI calculations.
- Table S7 – Toxicological thresholds for plastic additives (only compounds with a threshold defined were reported). Values in bold are those used in the risk assessment calculations.
- Table S8 – PAEs concentrations (ng/g) in sanitary pads, panty liners, tampons, reusable sanitary pads, menstrual underwear and menstrual cups.
- Table S9 – OPEs concentrations (ng/g) sanitary pads, panty liners, tampons, reusable sanitary pads, menstrual underwear and menstrual cups.
- Table S10 – APs concentrations (ng/g) in sanitary pads, panty liners, tampons, reusable sanitary pads, menstrual underwear and menstrual cups.
- Table S11 – Pairwise comparisons for total plastic additives concentrations (ng/g) in different menstrual products using Wilcoxon rank sum exact test (*=p<0.05).
- Table S12 – Pairwise comparisons for ∑PAEs concentrations (ng/g) in different menstrual products using Wilcoxon rank sum exact test (*=p<0.05).
- Table S13 – Pairwise comparisons for ∑OPEs concentrations (ng/g) in different menstrual products using Wilcoxon rank sum exact test (*=p<0.05)

- Table S14 – Pairwise comparisons for ∑APs concentrations (ng/g) in different menstrual products using Wilcoxon rank sum exact test (*=p<0.05)
- Table S15 – Concentrations (ng/product) of PAEs, OPEs and APs detected in the packaging of single use products. For sanitary pads the liner plastic (LP) and packing (P) were included, for panty liners only the liner plastic (LP) and for tampons the applicator (A) and packaging (P). Analysed plastic additives which are not reported in this table were not detected in any of the samples.
- Table S16 – Spearman's rank correlation coefficient between additives concentrations in products and packaging (DF=detection frequency).
- Table S17 - PAEs concentrations (ng/g) in sanitary pads from this study and different studies reported in the literature.
- Table S18 - PAEs concentrations (ng/g) in panty linters and tampons from this study and another study in the literature.
- Table S19 - Plastic additives emissions to the environment from menstrual products used by women that menstruate in Spain in kg/year.
- Table S20 – PAEs concentrations (ng/product) in sanitary pads, panty liners, tampons, reusable sanitary pads, menstrual underwear and menstrual cups.
- Table S21 – OPEs concentrations (ng/product) in sanitary pads, panty liners, tampons, reusable sanitary pads, menstrual underwear and menstrual cups.
- Table S22 – APs concentrations (ng/product) in sanitary pads, panty liners, tampons, reusable sanitary pads, menstrual underwear and menstrual cups.

1. Materials and methods

1.1. Chemicals and consumables

Acetone, hexane and formic acid were obtained from J.T. Baker (Center Valley, PA, USA). Methanol was purchased from Merck (Darmstadt, Germany). Ammonium acetate 98% was purchased from Sigma-Aldrich (St. Louis, MO, USA). Glass wool was provided by Panreac AppliChem (Barcelona, Spain). The full list of native and labelled standards used for plastic additives analysis is provided in Table S1.

Table S1 – Abbreviation, full name, CAS number and manufactures of plastic additives native and labelled standards. The last column for each analyte (ISTD) indicates the internal isotopically labelled standard used for quantification.

Abbreviation	Name	CAS number	Manufacturer	ISTD
			**PAEs**	
MEHP	Mono (2-ethylhexyl) phthalate	4376-20-9	AccuStandard (New Haven, CT, USA)	d4-MEHP
BBzP	Butyl benzyl phthalate	85-68-7	AccuStandard (New Haven, CT, USA)	d4-BBzP
DiBP	Diisobutyl phthalate	84-69-5	AccuStandard (New Haven, CT, USA)	d4-DiBP
DnBP	Di-n-butyl phthalate	84-74-2	AccuStandard (New Haven, CT, USA)	d4-DnBP
DHexP	Dihexyl phthalate	84-75-3	AccuStandard (New Haven, CT, USA)	d4-DHexP
DEHP	Di(2-ethylhexyl) phthalate	117-81-7	AccuStandard (New Haven, CT, USA)	d4-DEHP
DnOP	Di-n-octyl phthalate	117-84-0	AccuStandard (New Haven, CT, USA)	d4-DnOP
DiNP	Diisononyl phthalate	28553-12-0	AccuStandard (New Haven, CT, USA)	d4-DINP
DiDP	Diisodecyl phthalate	26761-40-0	AccuStandard (New Haven, CT, USA)	d4-DINP
			**OPEs**	
TEP	Triethyl phosphate	78-40-0	Merck (Darmstadt, Germany)	d15-TEP
TCEP	Tris(2-chloroethyl) phosphate	115-96-8	Santa Cruz Biotechnology (Santa Cruz, CA, USA)	d12-TCEP
TPPO	Triphenylphosphine oxide	791-28-6	Merck (Darmstadt, Germany)	d15-TDClPP
TCIPP	Tris(2-chloroisopropyl) phosphate	13674-84-5	Santa Cruz Biotechnology (Santa Cruz, CA, USA)	d15-TDClPP
TPrP	Tripropyl phosphate	513-08-6	Merck (Darmstadt, Germany)	d21-TPrP
TDCIPP	Tris(1,3-dichloro-2-propyl) phosphate	13674-87-8	Merck (Darmstadt, Germany)	d15-TDClPP
TPHP	Triphenyl phosphate	115-86-6	Merck (Darmstadt, Germany)	d15-TPHP
TNBP	Tri-n-butyl phosphate	126-73-8	Merck (Darmstadt, Germany)	d27-TNBP
DCP	Diphenyl cresyl phosphate	26444-49-5	Merck (Darmstadt, Germany)	d27-TNBP
TBOEP	Tris(2-butoxyethyl) phosphate	78-51-3	Santa Cruz Biotechnology (Santa Cruz, CA, USA)	13C2-TBOEP
RDP	Resorcinol bis(diphenyl phosphate)	57583-54-7	Cambridge Isotope Laboratories Inc. (Andover, MA, USA)	13C2-TBOEP
2IPPDPP	2-isopropylphenyl diphenyl phosphate	64532-94-1	Wellington Laboratories Inc. (Guelph, ON, Canada)	13C2-TBOEP
4IPPDPP	4-isopropylphenyl diphenyl phosphate	55864-04-5	Wellington Laboratories Inc. (Guelph, ON, Canada)	13C2-TBOEP
TCP	Tricresyl phosphate	1330-78-5	Dr. Ehrenstorfer (Augsburg, Germany)	13C2-TBOEP

EHDPP	2-ethylhexyl diphenyl phosphate	1241-94-7	AccuStandard (New Haven, CT, USA)	13C2-TBOEP
B4IPPPP	Bis(4-isopropylphenyl)phenyl phosphate	55864-07-8	Wellington Laboratories Inc. (Guelph, ON, Canada)	13C2-TBOEP
TEHP	Tris(2-ethylhexyl) phosphate	78-42-2	Santa Cruz Biotechnology (Santa Cruz, CA, USA)	d51-TEHP
		**APs**		
ATEC	Acetyl triethyl citrate	77-89-4	Dr. Ehrenstorfer (Augsburg, Germany)	d3-ATBC
DIPA	Diisopropyl adipate	6938-94-9	WAKO (Osaka, Japan)	d3-ATBC
TBC	Tributyl citrate	77-94-1	Dr. Ehrenstorfer (Augsburg, Germany)	d3-ATBC
DBA	Dibutyl adipate	105-99-7	Dr. Ehrenstorfer (Augsburg, Germany)	d3-ATBC
ATBC	Acetyl tributyl citrate	77-90-7	AccuStandard (New Haven, CT, USA)	d3-ATBC
DEHA	Di(2-ethylhexyl) adipate	103-23-1	AccuStandard (New Haven, CT, USA)	d8-DEHA
BTHC	n-butyryl-tri-n-hexyl citrate	82469-79-2	Dr. Ehrenstorfer (Augsburg, Germany)	d4-DnOP
DINA	Diisononyl adipate	33703-08-1	Dr. Ehrenstorfer (Augsburg, Germany)	d4-DnOP
DINCH	1,2-cyclohexanedicarboxylic acid, 1,2-diisononyl ester	166412-78-8	Dr. Ehrenstorfer (Augsburg, Germany)	d4-DnOP
		**Internal standards (ISTDs)**		
d4-MEHP	Mono (2-ethylhexyl) phthalate-d4	1276197-22-8	Dr. Ehrenstorfer (Augsburg, Germany)	
d4-BBzP	Butyl benzyl phthalate-d4	93951-88-3	Analytical Standard Solutions (Saint-Jean-d'Illac, France)	
d4-DiBP	Diisobutyl phthalate-d4	358730-88-8	AccuStandard (New Haven, CT, USA)	
d4-DnBP	Di-n-butyl phthalate-d4	93952-11-5	AccuStandard (New Haven, CT, USA)	
d4-DHexP	Dihexyl phthalate-d4	1015854-55-3	AccuStandard (New Haven, CT, USA)	
d4-DEHP	Di(2-ethylhexyl) phthalate-d4	93951-87-2	AccuStandard (New Haven, CT, USA)	
d4-DnOP	Di-n-octyl phthalate-d4	93952-13-7	AccuStandard (New Haven, CT, USA)	
d4-DINP	Diisononyl phthalate-d4	1202865-43-7	CDN Isotopes (Pointe-Claire, QC, Canada)	
d15-TEP	Triethyl phosphate-d15	135942-11-9	Cambridge Isotope Laboratories Inc. (Andover, MA, USA)	
d12-TCEP	Tris(2-chloroethyl) phosphate-d12	1276500-47-0	Cambridge Isotope Laboratories Inc. (Andover, MA, USA)	
d21-TPrP	Tripropyl phosphate-d21	1219794-92-9	Cambridge Isotope Laboratories Inc. (Andover, MA, USA)	
d15-TDCIPP	Tris(1,3-dichloro-2-propyl) phosphate-d15	1447569-77-8	Cambridge Isotope Laboratories Inc. (Andover, MA, USA)	
d15-TPHP	Triphenyl phosphate-d15	1173020-30-8	Cambridge Isotope Laboratories Inc. (Andover, MA, USA)	
d27-TNBP	Tri-n-butyl phosphate-d27	61196-26-7	Cambridge Isotope Laboratories Inc. (Andover, MA, USA)	
13C2-TBOEP	Tris(2-butoxy-[13C2]-ethyl) phosphate	-	Wellington Laboratories Inc. (Guelph, ON, Canada)	
d51-TEHP	Tris(2-ethylhexyl) phosphate-d51	1259188-37-8	Toronto Research Chemicals (Toronto, ON, Canada)	
d3-ATBC	Acetyl tributyl citrate-d3	1794753-49-3	CDN Isotopes (Pointe-Claire, QC, Canada)	
d8-DEHA	Di(2-ethylhexyl) adipate-d8	1214718-98-5	Chiron (Trondheim, Norway)	

91

1.2. Sample selection

Table S2 – Detailed information on the menstrual products included in this study (ND=no data). The product line is specified for products from the same brand, marketed with different name because of different properties, like scent and comfort.

Sample ID	Brand	Product line*	Size	Origin	Country (production)	Composition	Scent or perfume?	Unit price (€)	Number of uses
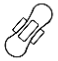 **Single use sanitary pads – SP (n=10)**									
SP-1	A	1	Normal	Supermarket	Spain	Polyolefins for the upper layer, absorbent cellulose with absorbent gel, rayon and polyester, polyolefins for the external layer, adhesives	Yes	0.16	1
SP-2	A	1	Super	Supermarket	Spain	Polyolefins for the upper layer, absorbent cellulose with absorbent gel, rayon and polyester, polyolefins for the external layer, adhesives	Yes	0.25	1
SP-3	A	1	Super plus	Supermarket	Spain	Polyolefins for the upper layer, absorbent cellulose with absorbent gel, rayon and polyester, polyolefins for the external layer, adhesives	Yes	0.28	1
SP-4	A	1	Night	Supermarket	Italy	Polyolefins for the upper layer, absorbent cellulose with absorbent gel, rayon and polyester, polyolefins for the external layer, adhesives	Yes	0.32	1
SP-5	A	1	Night extra	Supermarket	Italy	Polyolefins for the upper layer, absorbent cellulose with absorbent gel, rayon and polyester, polyolefins for the external layer, adhesives	Yes	0.31	1
SP-6	A	2	Normal	Supermarket	Italy	Polyolefins for the upper layer, absorbent sponge, polyolefins for the external layer, adhesives	ND	0.29	1
SP-7	A	3	Normal	Supermarket	Spain	Polyolefins for the upper layer, absorbent cellulose with absorbent gel, rayon and polyester, polyolefins for the external layer, adhesives	Yes	0.12	1
SP-8	B	-	Normal	Supermarket	Spain	Polyolefins for the upper layer, absorbent cellulose with absorbent gel, rayon and polyester, adhesives	Yes	0.13	1
SP-9	C	-	Normal	Supermarket	France	ND	ND	0.16	1
SP-10	D	-	Normal	Supermarket	France	ND	No	0.10	1
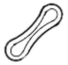 **Panty liners – PL (n=8)**									
PL-1	A	1	Normal	Supermarket	Ukraine	ND	ND	0.12	1

PL-2	A	2	Normal	Supermarket	Italy	ND	Yes	0.06	1
PL-3	B	-	Normal	Supermarket	Italy	ND	No	0.13	1
PL-4	C	-	Normal	Supermarket	Italy	ND	No	0.05	1
PL-5	D	-	Normal	Supermarket	Slovakia	ND	No	0.14	1
PL-6	E	1	Normal	Supermarket	Spain	ND	ND	0.04	1
PL-7	E	2	Normal	Supermarket	Spain	Top layer is 100% organic cotton	No	0.05	1
PL-8	F	-	Normal	Supermarket	France	Surface layer in non-woven fabric, cellulose fibers, superabsorbent (sodium polyacrylate), synthetic fibers (polyethylene, polypropylene, polyester, and elastomer, alone or mixed), adhesives (thermoplastic polymers)	ND	0.03	1
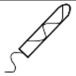 **Tampons – T (n=9)**									
T-1	A	1	Small	Supermarket	Italy	Absorbent made of cotton and/or rayon and textile layer made of rayon, polyester, polyethylene and polypropylene	No	0.18	1
T-2	A	1	Super	Supermarket	Italy	Absorbent made of cotton and/or rayon and textile layer made of rayon, polyester, polyethylene and polypropylene	No	0.22	1
T-3	A	1	Super plus	Supermarket	Italy	Absorbent made of cotton and/or rayon and textile layer made of rayon, polyester, polyethylene and polypropylene	No	0.25	1
T-4	A	2	Normal	Supermarket	Germany	Absorbent made of cotton and/or rayon and textile layer made of rayon, polyester, polyethylene and polypropylene	No	0.24	1
T-5	A	1	Normal	Supermarket	Germany	Absorbent made of cotton and/or rayon and textile layer made of rayon, polyester, polyethylene and polypropylene	No	0.17	1
T-6	A	3	Normal	Supermarket	Netherlands	Absorbent made of cotton and/or rayon and textile layer made of rayon, polyester, polyethylene and polypropylene	No	0.29	1
T-7	B	-	Normal	Supermarket	France	100% organic cotton with no scents. This product had no applicator.	No	0.17	1
T-8	C	-	Normal	Supermarket	France	ND	No	0.11	1
T-9	D	-	Normal	Supermarket	Israel	ND	No	0.11	1
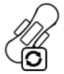 **Reusable sanitary pads – RSP (n=4)**									
RSP-1	A	-	M	Online	China	Lining 95% cotton and 5% elastane, middle: microfiber 100% polyester, breathable TPU, body: 95% cotton and 5% elastane	ND	35	N/D
RSP-2	B	-	M	Regional health initiative	Spain	Upper layer: 100% organic cotton, absorbent layer: 70% bamboo and 30% organic cotton, waterproof layer: PUL	ND	-	3-5 years use

						textile (Polyester water-repellent fabric, with TPU film), external layer: 100% cotton			
RSP-3	C	-	Day	Online	ND	External layers: bamboo fiber, absorbent layer: superabsorbent microfiber, waterproof layer: PUL textile (Polyester water-repellent fabric, with TPU film)	ND	12.9	5 years use
RSP-4	C	-	Night	Online	ND	External layers: bamboo fiber, absorbent layer: superabsorbent microfiber, waterproof layer: PUL textile (Polyester water-repellent fabric, with TPU film)	ND	13.9	5 years use
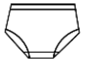 **Menstrual underwear – MU (n=4)**									
MU-1	A	-	M	Online	Vietnam	Body: 71% Polyamide, 29% Elastane/ Crotch: Face: 57% Polyester, 34% Elastomultiester, 9% Elastane/ Back: 71% Polyamide, 29% Elastane	ND	19.9	N/D
MU-2	B	-	M	Regional health initiative	Spain	Organic cotton	ND	-	100 uses
MU-3	C	-	M	Online	China	95% cotton, 5% elastane	ND	9	N/D
MU-4	D	-	M	Online	ND	External layer: 95% bamboo fiber and 5% spandex, internal layer: 80% polyester and 20% nylon, waterproof layer: PUL textile (Polyester water-repellent fabric, with TPU film)	ND	18.8	5 years use
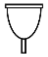 **Menstrual cups – MC (n=6)**									
MC-1	A	-	L	Supermarket	China	100% medical grade silicone	ND	5.90	N/D
MC-2	B	-	L	Online	Italy	100% medical grade silicone	ND	11.4	N/D
MC-3	C	-	L	Online	Denmark	100% medical grade silicone	ND	27.4	10 years use
MC-4	D	-	L	Regional health initiative	Spain	100% medical grade silicone	ND		N/D
MC-5	E	-	L	Online	Germany	Medical grade TPE (thermoplastic elastomer)	ND	16.9	At least 3 years use
MC-6	F	-	L	Online	Spain	Medical grade TPE (thermoplastic elastomer)	ND	14.9	N/D

*Product line= products from the same brand, marketed with different name because of different properties, like scent and comfort

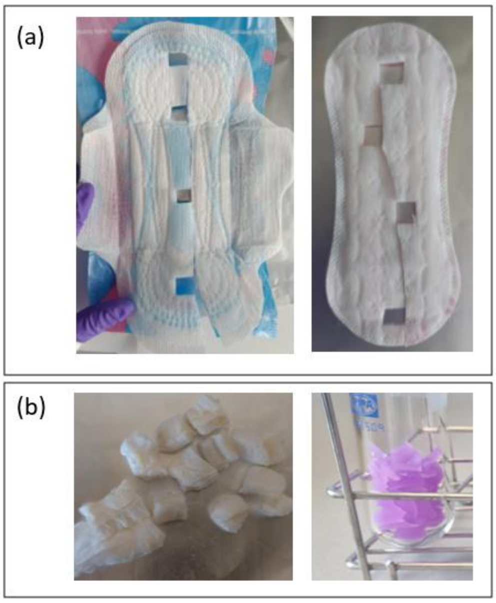

Figure S1 – Examples of selection of representative portions of menstrual products for analysis: (a) sanitary pads and panty liners; (b) tampons and menstrual cups.

1.3.Instrumental analysis

Plastic additives were analysed using an ultrahigh pressure liquid chromatography triple-quadrupole mass-spectrometry (UHPLC-MS/MS) instrument (Thermo Fisher Scientific, Waltham, MA, USA) comprising a PAL autosampler, an LC quaternary pump and a TSQ Quantiva triple quadrupole mass spectrometer with a heated-electrospray ionization source operating in positive mode. Chromatographic separation was achieved with a Purosphere Star RP-18 (125 mm × 0.2 mm, particle size 5 µm) column. The instrumental parameters for the chromatography and mass spectrometry methods are provided by Fernández-Arribas et al [1]. The instrumental method included 9 PAEs, 17 OPEs and 9 APs (Table S1).

1.4.Analytical method QA/QC

The analytical method was validated in terms of recovery, sensitivity and reproducibility. For this purpose, 0.1 g samples of 3 different types of products (sanitary pads, reusable sanitary

pads and menstrual cups) were spiked with 50 ng of native OPEs, 100 ng of PAEs, 1000 ng of ATBC and 100 ng of the other APs to determine recoveries and LODs. The samples were let equilibrate for at least 2 hours and extracted with the method previously described. The internal standard mixture was added to the extracts just before injection to allow quantification. These experiments were performed in triplicate to also allow the evaluation of reproducibility.

In addition, for sanitary pads, which presented an heterogenous composition, reproducibility of the method within the same product and within the same batch was evaluated. Reproducibility within the same product was evaluated preparing 3 different samples taking pieces from different parts of the sanitary pads. Reproducibility within the same batch was tested taking 3 different sanitary pads from the same product package sampling from the same parts of the product in all replicates.

Table S3 – Recovery values (%) for PAEs, OPEs and APs in sanitary pads, menstrual underwear and menstrual cups.

**Compound**	**Sanitary pads (n=3)** Average ± SD (RSD)	**Menstrual underwear (n=3)** Average ± SD (RSD%)	**Menstrual cups (n=3)** Average ± SD (RSD%)
**PAEs**			
MEHP	60 ± 11 (18%)	53 ± 5 (9%)	70 ± 3 (4%)
BBzP	62 ± 10 (16%)	57 ± 7 (12%)	97 ± 9 (9%)
DBP (DiBP+DnBP)	58 ± 16 (28%)	55 ± 9 (16%)	95 ± 10 (11%)
DHexP	55 ± 10 (18%)	66 ± 10 (15%)	73 ± 12 (16%)
DEHP	92 ± 14 (15%)	75 ± 8 (11%)	94 ± 9 (10%)
DnOP	63 ± 10 (16%)	59 ± 4 (7%)	63 ± 10 (16%)
DiNP	66 ± 12 (18%)	71 ± 3 (4%)	59 ± 11 (19%)
DiDP	54 ± 10 (19%)	59 ± 3 (5%)	58 ± 10 (17%)
**OPEs**			
TEP	45 ± 5 (11%)	47 ± 5 (11%)	71 ± 11 (15%)
TCEP	57 ± 7 (12%)	60 ± 2 (3%)	79 ± 5 (6%)
TPPO	60 ± 3 (5%)	73 ± 11 (15%)	75 ± 4 (5%)
TCIPP	76 ± 3 (4%)	71 ± 6 (8%)	81 ± 2 (2%)
TPrP	54 ± 5 (9%)	46 ± 2 (4%)	65 ± 4 (6%)
TDCIPP	60 ± 6 (10%)	68 ± 5 (7%)	82 ± 5 (6%)
TPHP	55 ± 7 (13%)	59 ± 9 (15%)	63 ± 3 (5%)
TNBP	56 ± 4 (7%)	83 ± 5 (6%)	68 ± 10 (15%)
DCP	59 ± 8 (14%)	77 ± 5 (6%)	66 ± 7 (11%)
TBOEP	53 ± 6 (11%)	79 ± 10 (13%)	79 ± 8 (10%)
RDP	46 ± 7 (15%)	68 ± 12 (18%)	63 ± 5 (8%)
2IPPDPP	59 ± 6 (10%)	61 ± 9 (15%)	54 ± 6 (11%)
4IPPDPP	44 ± 5 (11%)	57 ± 5 (9%)	60 ± 3 (5%)
TCP	48 ± 6 (13%)	57 ± 8 (14%)	56 ± 10 (18%)
EHDPP	54 ± 7 (13%)	64 ± 12 (19%)	61 ± 4 (7%)
B4IPPP	53 ± 5 (9%)	62 ± 10 (16%)	59 ± 4 (7%)
TEHP	64 ± 11 (17%)	57 ± 10 (18%)	60 ± 9 (15%)
**APs**			
ATEC	47 ± 3 (6%)	66 ± 4 (6%)	77 ± 7 (9%)
DIPA	48 ± 3 (6%)	59 ± 7 (12%)	71 ± 4 (6%)
TBC	93 ± 4 (4%)	72 ± 9 (13%)	74 ± 3 (4%)
DBA	65 ± 13 (20%)	68 ± 7 (10%)	54 ± 4 (7%)
ATBC	56 ± 4 (7%)	72 ± 6 (8%)	81 ± 4 (5%)
DEHA	54 ± 4 (7%)	71 ± 8 (11%)	105 ± 12 (11%)
BTHC	75 ± 7 (9%)	79 ± 15 (19%)	62 ± 8 (13%)
DINA	50 ± 6 (12%)	61 ± 10 (16%)	67 ± 10 (15%)
DINCH	61 ± 6 (10%)	58 ± 3 (5%)	55 ± 8 (15%)

Table S4 – LODs (ng/g) for PAEs, OPEs and APs in sanitary pads, menstrual underwear and menstrual cups.

Compound	Sanitary pads, panty liners and tampons	Menstrual underwear and reusable sanitary pads	Menstrual cups
**PAEs**			
MEHP	2.47	2.03	3.05
BBzP	1.25	2.38	1.94
DBP (DiBP+DnBP)	7.81	12.7	13.4
DHexP	0.72	0.80	0.80
DEHP	4.10	2.65	1.58
DnOP	3.41	2.91	3.23
DiNP	33.8	57.7	16.7
DiDP	71.9	24.0	31.8
**OPEs**			
TEP	4.74	7.63	2.40
TCEP	3.69	2.32	2.53
TPPO	0.06	0.49	0.48
TCIPP	0.56	0.63	0.45
TPrP	0.16	0.35	0.66
TDCIPP	0.86	0.12	0.33
TPHP	0.16	0.81	0.12
TNBP	0.18	0.75	0.29
DCP	0.60	0.31	0.19
TBOEP	0.29	0.10	0.72
RDP	0.06	0.23	0.17
2IPPDPP	0.08	0.67	0.09
4IPPDPP	0.15	0.24	0.07
TCP	0.15	0.09	0.13
EHDPP	1.02	1.17	1.12
B4IPPP	0.23	0.29	0.20
TEHP	12.5	9.13	6.01
**APs**			
ATEC	4.74	2.96	5.01
DIPA	2.48	2.56	1.22
TBC	0.94	4.32	0.83
DBA	11.4	13.1	20.1
ATBC	2.31	1.69	1.56
DEHA	40.4	54.1	33.3
BTHC	5.84	2.21	5.25
DINA	6.79	3.75	4.05
DINCH	93.4	61.4	50.4

Table S5 – Method reproducibility within the same product and within the same batch for sanitary pads (n=3). Reproducibility can be reported only for compounds detected in the samples analysed.

Compound	RSD % within the same product	RSD % within the same batch
DiNP	4	15
TPPO	2	17
TCIPP	10	12
TDCIPP	5	20
TPHP	9	13
TNBP	5	4
DCP	5	8
RDP	13	10
TBC	9	22
ATBC	6	12

1.5. Dermal exposure calculations and human health risk assessment

Table S6 – Exposure parameters values used for EDI calculations.

	Sanitary pads	Panty liners	Tampons	Reusable sanitary pads	Menstrual underwear	Menstrual cups
N [2]	6	4	6	4	4	1
NU [3]	1	1	1	252	252	503
ERF	1*					
AF	1*					
BW [4], [5]	51.0 (12-18 years old), 63.9 (19-40 years old), 69.6 (41-51 years old)					

*worst case scenario assumption

Table S7 – Toxicological thresholds for plastic additives (only compounds with a threshold defined were reported). Values in bold are those used in the risk assessment calculations.

Compound	Chronic RfD (ng/kg bw/day)	MRLs (ng/kg bw/day)	TDI EFSA (ng/kg bw/day)	SFO $(mg/kg\ bw/day)^{-1}$
**PAEs**				
BBzP	**$2.00*10^5$ [6]**	N/D	N/D	**$1.9 * 10^{-3}$ [7]**
DnBP	**$1.00*10^5$ [6]**	$5.00*10^5$ (Acute) [8]	N/D	N/D
DEHP	**$2.00*10^4$ [6]**	$3.00*10^3$ (Acute) [8]<br>$1.00*10^2$ (Intermediate) [8]	N/D	**$1.4 * 10^{-2}$ [6]**
DiNP	N/D	N/D	**$1.50*10^5$ [9]**	N/D
DiDP	N/D	N/D	**$1.50*10^5$ [9]**	N/D
**OPEs**				
TCEP	**$7.00*10^3$ [7]**	$6.00*10^5$ (Intermediate) [8]<br>$2.00*10^5$ (Chronic) [8]	N/D	**$2.0*10^{-2}$ [7]**
TPPO	**$2.00*10^4$ [7]**	N/D	N/D	N/D
TCIPP	**$1.00*10^4$ [7]**	N/D	N/D	N/D
TDCIPP	N/D	$5.00*10^4$ (Intermediate) [8]<br>**$2.00*10^4$ (Chronic) [8]**	N/D	N/D
TPHP	**$7.00*10^3$ [10]**	N/D	N/D	N/D
TNBP	**$1.00*10^4$ [7]**	$1.10*10^6$ (Acute) [8]<br>$8.00*10^4$ (Intermediate) [8]<br>$8.00*10^4$ (Chronic) [8]	N/D	**$9.0*10^{-3}$ [7]**
TBOEP	N/D	$4.80*10^6$ (Acute) [8]<br>**$9.00*10^4$ (Intermediate) [8]**	N/D	N/D
TCP	N/D	$4.00*10^4$ (Intermediate) [8]<br>**$2.00*10^4$ (Chronic) [8]**	N/D	N/D
EHDPP	**$6.00*10^2$ [11]**	N/D	N/D	N/D
TEHP	**$1.00*10^5$ [7]**	N/D	N/D	**$3.2*10^{-3}$ [7]**
**APs**				
ATBC	N/D	N/D	**$1.00*10^6$[12]**	N/D
DEHA	$6.00*10^5$ [6]	N/D	**$3.00*10^5$ [13]**	**$1.2*10^{-3}$ [6]**
DINCH	N/D	N/D	**$1.00*10^6$ [12]**	N/D

Table S8 – PAEs concentrations (ng/g) in sanitary pads, panty liners, tampons, reusable sanitary pads, menstrual underwear and menstrual cups.

	Sanitary pads (n= 10)		Panty liners (n= 8)		Tampons (n= 9)		Reusable sanitary pads (n= 4)		Menstrual underwear (n= 4)		Menstrual cups (n= 6)	
**Compound**	DF (%)	Median; Mean<br>Range	DF (%)	Median; Mean<br>Range	DF (%)	Median; Mean<br>Range	DF (%)	Median; Mean<br>Range	DF (%)	Median; Mean<br>Range	DF (%)	Median; Mean<br>Range
MEHP	0	<LOD; <LOD<br><LOD	0	<LOD; <LOD<br><LOD	0	<LOD; <LOD<br><LOD	0	<LOD; <LOD<br><LOD	0	<LOD; <LOD<br><LOD	0	<LOD; <LOD<br><LOD
BBzP	0	<LOD; <LOD<br><LOD	25	<LOD; 3.31<br><LOD-17.6	11	<LOD; <LOD<br><LOD-3.90	0	<LOD; <LOD<br><LOD	0	<LOD; <LOD<br><LOD	0	<LOD; <LOD<br><LOD
DBP	0	<LOD; <LOD<br><LOD	37	<LOD; 209<br><LOD-1010	0	<LOD; <LOD<br><LOD	25	<LOD; 40.1<br><LOD-133	25	<LOD; 85.4<br><LOD-315	83	138; 149<br><LOD-275
DHexP	0	<LOD; <LOD<br><LOD	0	<LOD; <LOD<br><LOD	0	<LOD; <LOD<br><LOD	0	<LOD; <LOD<br><LOD	0	<LOD; <LOD<br><LOD	0	<LOD; <LOD<br><LOD
DEHP	10	<LOD; 121<br><LOD-1183	37	<LOD; 120<br><LOD-490	11	<LOD; 14.5<br><LOD-107	100	22825; 22518<br>4913-41929	50	161; 181<br><LOD-400	100	116; 264<br>36.1-1003
DnOP	0	<LOD; <LOD<br><LOD	0	<LOD; <LOD<br><LOD	0	<LOD; <LOD<br><LOD	0	<LOD; <LOD<br><LOD	0	<LOD; <LOD<br><LOD	0	<LOD; <LOD<br><LOD
DiNP	40	<LOD; 518<br><LOD-2591	25	<LOD; 285<br><LOD-1440	22	<LOD; 118<br><LOD-452	25	<LOD; 3564<br><LOD-14135	25	<LOD; 550<br><LOD-2077	33	<LOD, 479<br><LOD-1477
DiDP	40	<LOD; 723<br><LOD-4263	0	<LOD; <LOD<br><LOD	0	<LOD; <LOD<br><LOD	100	91.1; 107<br>54.7-192	0	<LOD; <LOD<br><LOD	50	<LOD; <LOD<br><LOD-53.5
**∑PAEs**	**60**	**835; 1368<br><LOD-5424**	**50**	**160; 668<br><LOD-1990**	**33**	**<LOD; 190<br><LOD-616**	**100**	**28856; 26231<br>5019-42193**	**50**	**269; 835<br><LOD-2731**	**100**	**852; 924<br>140-2031**

DF=detection frequency

Table S9 – OPEs concentrations (ng/g) sanitary pads, panty liners, tampons, reusable sanitary pads, menstrual underwear and menstrual cups.

**Compound**	**Sanitary pads (n= 10)**		**Panty liners (n= 8)**		**Tampons (n= 9)**		**Reusable sanitary pads (n= 4)**		**Menstrual underwear (n= 4)**		**Menstrual cups (n= 6)**	
	DF (%)	Median; Mean<br>Range	DF (%)	Median; Mean<br>Range	DF (%)	Median; Mean<br>Range	DF (%)	Median; Mean<br>Range	DF (%)	Median; Mean<br>Range	DF (%)	Median; Mean<br>Range
TEP	20	<LOD; 17.8<br><LOD-122	25	<LOD; 9.88<br><LOD-29.7	33	<LOD; 5.51<br><LOD-14.1	0	<LOD; <LOD<br><LOD	0	<LOD; <LOD<br><LOD	17	<LOD; 16.2<br><LOD-88.9
TCEP	0	<LOD; <LOD<br><LOD	0	<LOD; <LOD<br><LOD	100	24.6; 29.7<br>11.7-82.5	0	<LOD; <LOD<br><LOD	25	<LOD; 55.3<br><LOD-216	0	<LOD; <LOD<br><LOD
TPPO	10	<LOD; 2.04<br><LOD-20.0	50	7.33; 22.5<br><LOD-84.9	0	<LOD; <LOD<br><LOD	0	<LOD; <LOD<br><LOD	0	<LOD; <LOD<br><LOD	0	<LOD; <LOD<br><LOD
TCIPP	0	<LOD; <LOD<br><LOD	25	<LOD; 38.9<br><LOD-177	0	<LOD; <LOD<br><LOD	0	<LOD; <LOD<br><LOD	25	<LOD; 56.4<br><LOD-225	0	<LOD; <LOD<br><LOD
TPrP	0	<LOD; <LOD<br><LOD	0	<LOD; <LOD<br><LOD	0	<LOD; <LOD<br><LOD	0	<LOD; <LOD<br><LOD	0	<LOD; <LOD<br><LOD	0	<LOD; <LOD<br><LOD
TDCIPP	10	<LOD; 7.73<br><LOD-71.9	50	41.2; 85.1<br><LOD; 277	0	<LOD; <LOD<br><LOD	0	<LOD; <LOD<br><LOD	0	<LOD; <LOD<br><LOD	0	<LOD; <LOD<br><LOD
TPHP	30	<LOD; 2.33<br><LOD-11.5	37	<LOD; 2.13<br><LOD-9.88	33	<LOD; 0.24<br><LOD-0.58	100	820; 955<br>141-2040	100	316; 491<br>8.40-1325	0	<LOD; <LOD<br><LOD
TNBP	100	236; 217<br>110-319	75	22.4; 46.8<br><LOD-193	78	11.1; 21.2<br><LOD-99.7	0	<LOD; <LOD<br><LOD	0	<LOD; <LOD<br><LOD	0	<LOD; <LOD<br><LOD
DCP	30	<LOD; 2.02<br><LOD-8.48	50	1.43; 3.10<br><LOD-10.9	0	<LOD; <LOD<br><LOD	0	<LOD; <LOD<br><LOD	0	<LOD; <LOD<br><LOD	0	<LOD; <LOD<br><LOD
TBOEP	0	<LOD; <LOD<br><LOD	12	<LOD; 2.46<br><LOD-18.3	0	<LOD; <LOD<br><LOD	0	<LOD; <LOD<br><LOD	0	<LOD; <LOD<br><LOD	0	<LOD; <LOD<br><LOD
RDP	10	<LOD; 0.07<br><LOD-0.34	12	<LOD; 0.15<br><LOD-0.92	0	<LOD; <LOD<br><LOD	0	<LOD; <LOD<br><LOD	0	<LOD; <LOD<br><LOD	0	<LOD; <LOD<br><LOD
2IPPDPP	10	<LOD; 0.62<br><LOD-5.69	25	<LOD; 0.99<br><LOD-5.38	0	<LOD; <LOD<br><LOD	0	<LOD; <LOD<br><LOD	0	<LOD; <LOD<br><LOD	0	<LOD; <LOD<br><LOD
4IPPDPP	10	<LOD; 14.8<br><LOD-147	25	<LOD; 0.43<br><LOD-2.18	0	<LOD; <LOD<br><LOD	0	<LOD; <LOD<br><LOD	0	<LOD; <LOD<br><LOD	0	<LOD; <LOD<br><LOD
TCP	0	<LOD; <LOD<br><LOD	25	<LOD; 0.42<br><LOD-2.15	0	<LOD; <LOD<br><LOD	0	<LOD; <LOD<br><LOD	0	<LOD; <LOD<br><LOD	0	<LOD; <LOD<br><LOD
EHDPP	0	<LOD; <LOD<br><LOD	50	2.90; 4.80<br><LOD-16.7	11	<LOD; 1.70<br><LOD-9.52	75	322; 358<br><LOD-787	0	<LOD; <LOD<br><LOD	0	<LOD; <LOD<br><LOD
B4IPPP	0	<LOD; <LOD<br><LOD	12	<LOD; 0.38<br><LOD-1.90	0	<LOD; <LOD<br><LOD	0	<LOD; <LOD<br><LOD	0	<LOD; <LOD<br><LOD	0	<LOD; <LOD<br><LOD
TEHP	40	<LOD; 11.7<br><LOD-19.8	25	<LOD; 12.5<br><LOD-33.4	0	<LOD; <LOD<br><LOD	75	687; 686<br><LOD-1364	25	<LOD; 9.09<br><LOD-17.0	0	<LOD; <LOD<br><LOD
**∑OPEs**	**100**	**280; 280<br>141-517**	**100**	**117; 233<br>42.8-841**	**100**	**53.5; 69.2<br>31.4-208**	**100**	**1906; 2009<br>158-4068**	**100**	**558; 621<br>25.5-1342**	**17**	**<LOD; 25.3<br><LOD-98.0**

Table S10 – APs concentrations (ng/g) in sanitary pads, panty liners, tampons, reusable sanitary pads, menstrual underwear and menstrual cups.

	**Sanitary pads (n= 10)**		**Panty liners (n= 8)**		**Tampons (n= 9)**		**Reusable sanitary pads (n= 4)**		**Menstrual underwear (n= 4)**		**Menstrual cups (n= 6)**	
**Compound**	DF (%)	Median; Mean<br>Range	DF (%)	Median; Mean<br>Range	DF (%)	Median; Mean<br>Range	DF (%)	Median; Mean<br>Range	DF (%)	Median; Mean<br>Range	DF (%)	Median; Mean<br>Range
ATEC	0	<LOD; <LOD<br><LOD	0	<LOD; <LOD<br><LOD	0	<LOD; <LOD<br><LOD	0	<LOD; <LOD<br><LOD	0	<LOD; <LOD<br><LOD	0	<LOD; <LOD<br><LOD
DIPA	10	<LOD; 2.95<br><LOD-13.8	87	20.8; 16.9<br><LOD-28.6	33	<LOD; 2.72<br><LOD-5.59	0	<LOD; <LOD<br><LOD	0	<LOD; <LOD<br><LOD	0	<LOD; <LOD<br><LOD
TBC	100	21.5; 20.7<br>6.09-36.0	62	2.49; 50.9<br><LOD-253	44	<LOD; 1.19<br><LOD-3.27	25	<LOD; 55.2<br><LOD-212	25	<LOD; 7.77<br><LOD-21.9	0	<LOD; <LOD<br><LOD
DBA	0	<LOD; <LOD<br><LOD	37	<LOD; 13.4<br><LOD-27.7	0	<LOD; <LOD<br><LOD	0	<LOD; <LOD<br><LOD	0	<LOD; <LOD<br><LOD	17	<LOD; 73.4<br><LOD-369
ATBC	100	6748; 7296<br>2714-11314	75	136; 4048<br><LOD-13563	78	24.2; 62.5<br><LOD-205	25	<LOD; 27.0<br><LOD-105	0	<LOD; <LOD<br><LOD	100	36.8; 48.6<br>5.60-113
DEHA	30	<LOD; 932<br><LOD-6020	0	<LOD; <LOD<br><LOD	0	<LOD; <LOD<br><LOD	100	886; 877<br>71.3-1663	100	233; 635<br>148-1926	50	43.8; 63.1<br><LOD-164
BTHC	0	<LOD; <LOD<br><LOD	0	<LOD; <LOD<br><LOD	0	<LOD; <LOD<br><LOD	0	<LOD; <LOD<br><LOD	0	<LOD; <LOD<br><LOD	0	<LOD; <LOD<br><LOD
DINA	10	<LOD, 5.53<br><LOD-12.1	37	<LOD; 18.1<br><LOD-104	0	<LOD; <LOD<br><LOD	0	<LOD; <LOD<br><LOD	0	<LOD; <LOD<br><LOD	0	<LOD; <LOD<br><LOD
DINCH	0	<LOD; <LOD<br><LOD	37	<LOD; 263<br><LOD-1522	22	<LOD; 94.8<br><LOD-293	0	<LOD; <LOD<br><LOD	0	<LOD; <LOD<br><LOD	33	<LOD; 71.1<br><LOD-228
**∑APs**	**100**	**8873; 8331<br>2839-11455**	**100**	**319; 4439<br>137-13857**	**100**	**145; 203<br>113-525**	**100**	**999; 1016<br>341-1725**	**100**	**304; 701<br>210-1988**	**100**	**242; 260<br>95.6-438**

DF=detection frequency

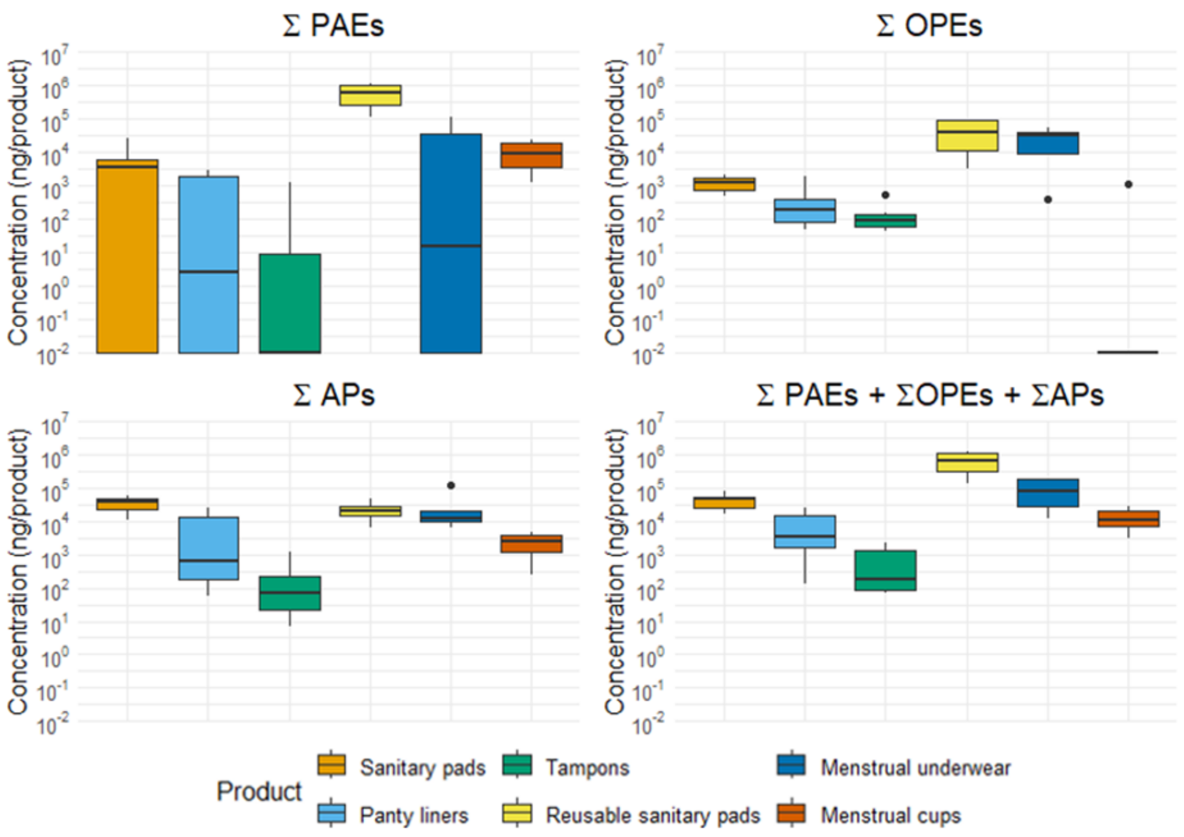

Figure S2 - ∑PAEs, ∑OPEs, ∑APs and total plastic additives concentrations (∑PAEs + ∑OPEs +∑APs) (ng/product) in sanitary pads, panty liners, tampons, reusable sanitary pads, menstrual underwear and menstrual cups (note the log scale).

Table S11 − Pairwise comparisons for total plastic additives concentrations (ng/g) in different menstrual products using Wilcoxon rank sum exact test (*=p<0.05)

	Sanitary pads	Panty liners	Tampons	Reusable sanitary pads	Menstrual underwear	Menstrual cups
Sanitary pads	-	-	-	-	-	-
Panty liners	0.104	-	-	-	-	-
Tampons	**0.000***	**0.024***	-	-	-	-
Reusable sanitary pads	0.103	**0.047***	**0.014***	-	-	-
Menstrual underwear	**0.015***	0.808	**0.045***	**0.048***	-	-
Menstrual cups	**0.002***	0.477	**0.048***	**0.024***	0.510	-
Median concentration (ng/g)	10014	2075	263	31856	1960	1116

Table S12 − Pairwise comparisons for ∑PAEs concentrations (ng/g) in different menstrual products using Wilcoxon rank sum exact test (*=p<0.05)

	Sanitary pads	Panty liners	Tampons	Reusable sanitary pads	Menstrual underwear	Menstrual cups
Sanitary pads	-	-	-	-	-	-
Panty liners	0.798	-	-	-	-	-
Tampons	0.151	0.510	-	-	-	-
Reusable sanitary pads	**0.036***	**0.036***	**0.036***	-	-	-
Menstrual underwear	0.700	0.928	0.700	0.074	-	-
Menstrual cups	0.700	0.403	**0.038***	**0.036***	0.700	-
Median concentration (ng/g)	835	160	<LOD	28856	269	852

Table S13 − Pairwise comparisons for ∑OPEs concentrations (ng/g) in different menstrual products using Wilcoxon rank sum exact test (*=p<0.05)

	Sanitary pads	Panty liners	Tampons	Reusable sanitary pads	Menstrual underwear	Menstrual cups
Sanitary pads	-	-	-	-	-	-
Panty liners	0.159	-	-	-	-	-
Tampons	**0.001***	0.189	-	-	-	-
Reusable sanitary pads	0.159	0.091	**0.026***	-	-	-
Menstrual underwear	0.277	0.367	0.248	0.367	-	-
Menstrual cups	**0.009***	**0.026***	0.057	**0.027***	**0.042***	-
Median concentration (ng/g)	280	117	53.5	1906	558	<LOD

165 **Table S14 –** Pairwise comparisons for ∑APs concentrations (ng/g) in different menstrual products using
166 Wilcoxon rank sum exact test (*=$p<0.05$)

	Sanitary pads	Panty liners	Tampons	Reusable sanitary pads	Menstrual underwear	Menstrual cups
Sanitary pads	-	-	-	-	-	-
Panty liners	0.272	-	-	-	-	-
Tampons	**0.000***	0.068	-	-	-	-
Reusable sanitary pads	**0.007***	0.653	**0.017***	-	-	-
Menstrual underwear	**0.007***	1.000	0.071	0.468	-	-
Menstrual cups	**0.002***	0.614	0.271	0.071	0.653	-
Median concentration (ng/g)	8873	319	145	999	304	242

167

168

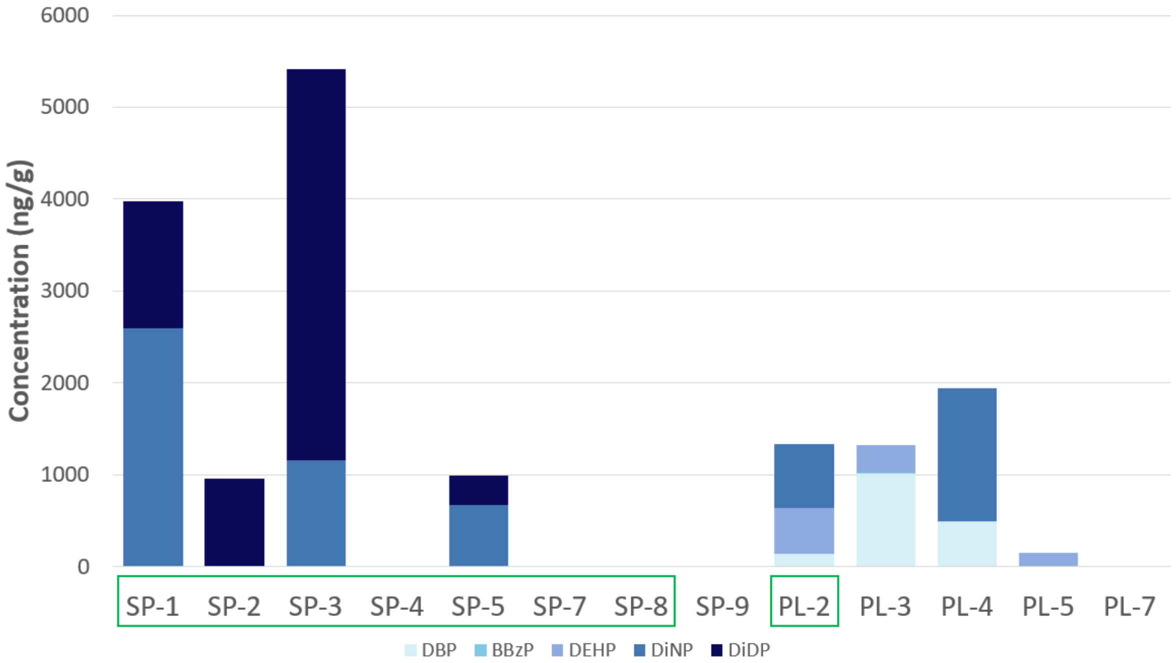

Figure S3 – PAEs concentrations (ng/g) in scented (highlighted in green) and unscented products.

Table S15 – Concentrations (ng/product) of PAEs, OPEs and APs detected in the packaging of single use products. For sanitary pads the liner plastic (LP) and packing (P) were included, for panty liners only the liner plastic (LP) and for tampons the applicator (A) and packaging (P). Analysed plastic additives which are not reported in this table were not detected in any of the samples.

	**Sanitary pads**				**Panty liners**		**Tampons**			
**Compound**	**LP**		**P**		**LP**		**A**		**P**	
	DF (%)	Median; Mean<br>Range	DF (%)	Median; Mean<br>Range	DF (%)	Median; Mean<br>Range	DF (%)	Median; Mean<br>Range	DF (%)	Median; Mean<br>Range
TEP	0	<LOD; <LOD<br><LOD	10	<LOD; 6.14<br><LOD-61.4	0	<LOD; <LOD<br><LOD	0	<LOD; <LOD<br><LOD	0	<LOD; <LOD<br><LOD
TCIPP	0	<LOD; <LOD<br><LOD	30	<LOD; 51.7<br><LOD-439	0	<LOD; <LOD<br><LOD	0	<LOD; <LOD<br><LOD	0	<LOD; <LOD<br><LOD
TPHP	0	<LOD; <LOD<br><LOD	100	10.9; 28.7<br>4.20-141	37	<LOD; 1.51<br><LOD-8.17	78	1.16; 1.28<br><LOD-3.52	89	4.54; 9.06<br><LOD-23.8
TNBP	70	9.03; 12.7<br><LOD-12.7	100	1074; 1181<br>102-3016	37	<LOD; 983<br><LOD-7663	67	81.2; 112<br><LOD-307	89	82.1; 126<br><LOD-410
TEHP	0	<LOD; <LOD<br><LOD	40	<LOD; <LOD<br><LOD-2.29	0	<LOD; <LOD<br><LOD	0	<LOD; <LOD<br><LOD	0	<LOD; <LOD<br><LOD
**∑OPEs**	**70**	**9.03; 12.7<br><LOD-12.7**	**100**	**1218; 1268<br>243-3089**	**37**	**<LOD; 986<br><LOD-7666**	**78**	**81.7; 113<br><LOD-310**	**89**	**103; 137<br><LOD-433**
DBP	0	<LOD; <LOD<br><LOD	60	40.9; 336<br><LOD-2834	50	18.7; 73.3<br><LOD-458	33	<LOD; 26.4<br><LOD-134	44	<LOD; 26.7<br><LOD-86.1
DEHP	0	<LOD; <LOD<br><LOD	70	116; 133<br><LOD-407	12	<LOD, 7.99<br><LOD-64.0	22	<LOD; 59.1<br><LOD-237	66	46.2; 51.4<br><LOD-137
DiNP	30	<LOD; 50.7<br><LOD-297	40	<LOD; 2668<br><LOD-17607	37	<LOD; 393<br><LOD-2368	55	33.2; 682<br><LOD-5077	78	41.9; 275<br><LOD-2222
DiDP	30	<LOD, 58.9<br><LOD-559	100	272; 1432<br>57.3-7985	12	<LOD; <LOD<br><LOD-109	33	<LOD; 29.2<br><LOD-159	66	12.6; 48.3<br><LOD-334
**∑PAEs**	**40**	**<LOD; 110<br><LOD-559**	**100**	**1301; 4569<br>109-20053**	**50**	**19.7: 488<br><LOD-2826**	**89**	**159; 797<br><LOD-5403**	**89**	**148; 401<br><LOD-2387**
DIPA	20	<LOD; <LOD<br><LOD-3.95	0	<LOD; <LOD<br><LOD	37	<LOD; 6.96<br><LOD-23.3	55	9.76; 15.5<br><LOD-55.4	22	<LOD; <LOD<br><LOD-9.37
TBC	40	<LOD; LOD<br><LOD-7.90	100	38.3; 75.4<br>8.03-254	37	<LOD; 326<br><LOD-1436	100	150; 308<br>2.47-1267	100	1209; 1109<br>4.46-3255
DBA	0	<LOD; <LOD<br><LOD	0	<LOD; <LOD<br><LOD	37	<LOD; 109<br><LOD-342	55	68.9; 85.9<br><LOD-291	89	33.2; 39.1<br><LOD-84.7

ATBC	100	164; 361<br>119-1248	100	1685; 12975<br>1568-82213	100	50.8; 25463<br>6.47-85461	100	9026; 12164<br>96.8-43396	100	13175; 11719<br>105-37107
DEHA	20	<LOD-21.7<br><LOD-199	80	482; 1801<br><LOD-11172	25	<LOD; 218<br><LOD-1396	44	<LOD; 87.4<br><LOD-577	100	33.5; 113<br>13.1-592
DINCH	0	<LOD; <LOD<br><LOD	0	<LOD; <LOD<br><LOD	20	<LOD; 187<br><LOD-1469	0	<LOD; <LOD<br><LOD	44	<LOD; 2192<br><LOD-19160
**∑APs**	**100**	**170; 386**<br>**119-1256**	**100**	**5439; 14891**<br>**1634-82783**	**100**	**51.6; 26310**<br>**6.47-90123**	**100**	**9328; 12750**<br>**265-45094**	**100**	**16513; 15174**<br>**263-40713**

Table S16 – Spearman's rank correlation coefficient between additives concentrations in products and packaging (DF=detection frequency).

	**Sanitary pads**				**Panty liners**				**Tampons**			
	DF product	DF packaging	Correlation	p-value	DF product	DF packaging	Correlation	p-value	DF product	DF packaging	Correlation	p-value
TNBP	100	100	0.58	0.088	75	33	NA	NA	78	77	-0.45	0.230
TBC	100	40	0.22	0.537	62	100	0.87	0.004	44	37	NA	NA
ATBC	100	100	0.36	0.313	75	100	0.85	0.007	78	100	0.60	0.086

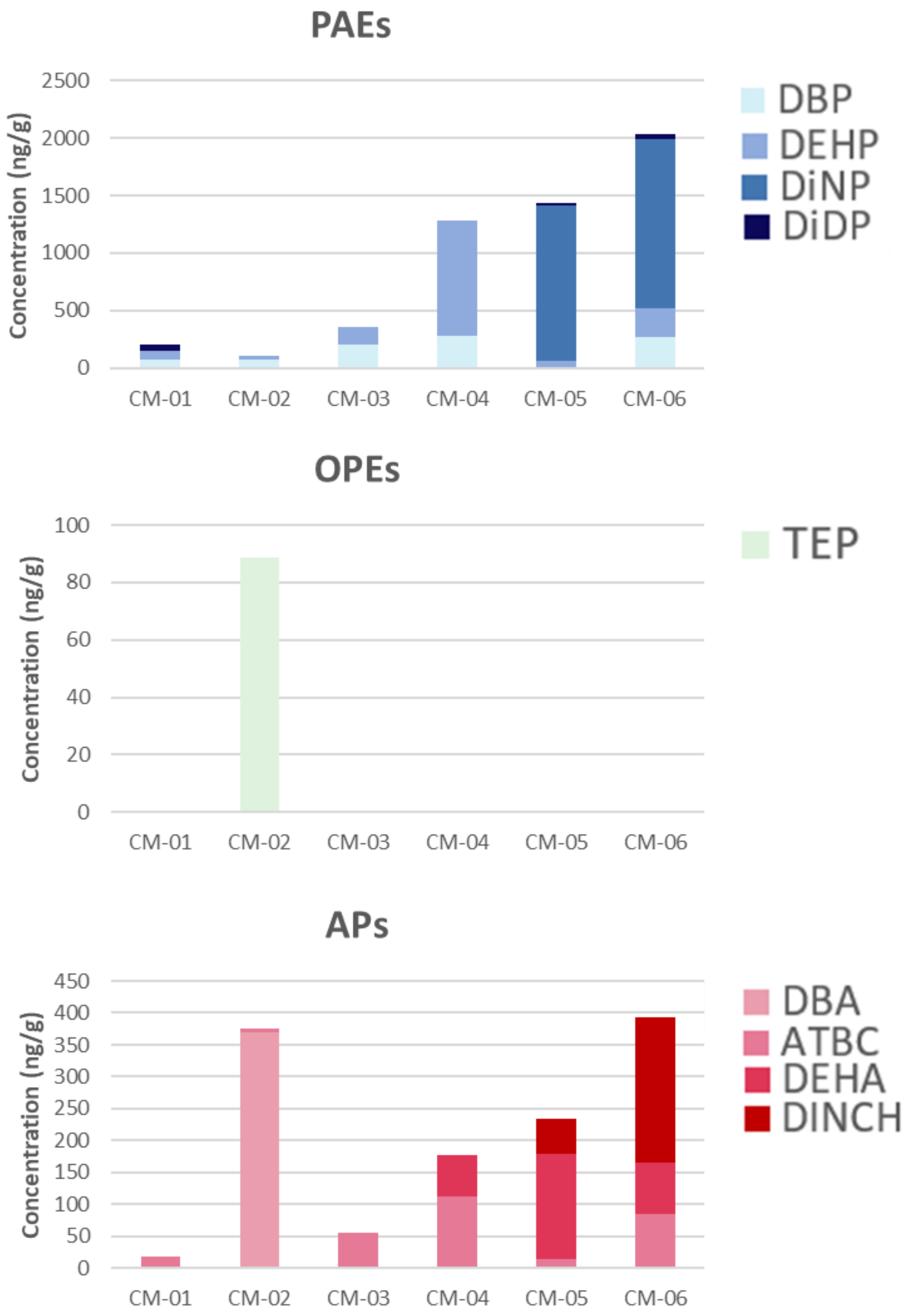

Figure S4 – Plastic additives concentrations (ng/g) in menstrual cups. Samples from CM-01 to CM-04 are silicone menstrual cups and samples CM-5 and CM-06 are TPE menstrual cups.

Table S17 - PAEs concentrations (ng/g) in sanitary pads from this study and different studies reported in the literature.

	This study	USA [14]	Japan [15]	South Korea [8]	USA [15]	UK [8]	Australia [15]	Germany [15]	China [16]
Sampling	2024	2019	2016	2016	2016	2016	2016	2016	2017-2018
DMP	-	81.5 (56.0-174)	165 (61.8-195)	124 (88.3-152)	124 (23.6-184)	210 (170-246)	175 (117-228)	237 (154-287)	<LOD (<LOD-430)
DEP	-	82.0 (50.9-1200)	67.3 (53.6-141)	76.3 (48.2-99.6)	126 (67.4-222)	99 (62.2-171)	73.7 (49.3-498)	155 (78.2-257)	<LOD (<LOD-870)
DiBP	<LOD	73.0 (25.9-5400)	580 (19.6-3331)	435 (290-3302)	764 (153-1652)	1008 (858-1282)	1024 (574-6193)	1424 (952-2439)	160 (<LOD-950)
DnBP		83.3 (22.0-3630)	804 (647-1194)	485 (412-2545)	470 (109-832)	731 (642-1172)	630 (501-1792)	909 (741-1591)	130 (<LOD-910)
DMEP	-	-	<LOD	<LOD (<LOD-20.4)	<LOD (<LOD-111)	<LOD (<LOD-176)	<LOD	<LOD (<LOD-200)	-
DPP	-	-	3.05 (<LOD-17.5)	14.6 (<LOD-296)	42.1 (<LOD-117)	15.8 (<LOD-74.4)	<LOD (<LOD-109)	35.5 (<LOD-457)	-
DEEP	-	-	<LOD (<LOD-110)	2.73 (<LOD-76.7)	34.8 (12.9-216)	25.3 (2.25-70.5)	29.3 (<LOD-116)	23.8 (<LOD-95.5)	-
BBzP	<LOD	<LOD	13.4 (<LOD-258)	27.8 (<LOD-107)	68.1 (<LOD-413)	64.1 (<LOD-198)	40.0 (<LOD-218)	109 (<LOD-222)	10.0 (<LOD-490)
DCHP	-	-	46.6 (<LOD-106)	<LOD	<LOD	<LOD	<LOD (<LOD-18.2)	<LOD (<LOD-2.86)	60.0 (<LOD-10800)
BMPP	-	-	138 (24.9-415)	77.4 (88.3-152)	199 (12.5-2465)	51.4 (19.0-262)	86.8 (5.86-580)	104 (28.1-992)	-
DBEP	-	-	183 (<LOD-633)	59.6 (48.2-99.6)	10.1 (<LOD-996)	8.94 (<LOD-151)	24.2 (<LOD-229)	21.6 (<LOD-53.4)	-
DEHP	<LOD (<LOD-1183)	38.7 (14.9-858)	1440 (928-7152)	1028 (290-3302)	822 (233-1683)	583 (480-798)	847 (636-2424)	664 (563-1515)	510 (<LOD-5850)
DnOP	<LOD (<LOD)	-	7.80 (<LOD-29.7)	<LOD	<LOD	<LOD (<LOD-9.08)	<LOD (<LOD-10.1)	12.0 (<LOD-122)	<LOD
DiNP	<LOD (<LOD-2590)	-	96.3 (<LOD-1091)	144 (<LOD-390)	<LOD (<LOD-75.7)	2.10 (<LOD-123)	89.8 (<LOD-242)	2.33 (<LOD-224)	-
DiDP	<LOD (<LOD-4263)	-	20 (<LOD-75.8)	16.6 (<LOD-63.0)	11.8 (<LOD-133)	22.7 (<LOD-118)	6.24 (<LOD-163)	4.07 (<LOD-164)	-
∑PAEs	789 (<LOD-5414)	362 (205-11200)	3643 (2643-11942)	2630 (2045-7893)	2922 (1733-4987)	3093 (2669-3871)	3981 (2424-9386)	4573 (2966-6089)	960 (140-11700)

Table S18 - PAEs concentrations (ng/g) in panty linters and tampons from this study and another study in the literature.

	Panty liners		Tampons	
	This study	USA [14]	This study	USA [14]
Sampling	2024	2019	2024	2019
DMP	-	249 (57.2-522)	-	214 (141-1650)
DEP	-	386 (45.6-1070)	-	190 (127-262)
DiBP	<LOD (<LOD-1010)	299 (25.1-5500)	<LOD	99.2 (57.9-326)
DnBP		393 (21.3-6070)		125 (72.0-2240)
BBzP	<LOD (<LOD-17.6)	<LOD	<LOD (<LOD-3.90)	<LOD
DEHP	<LOD (<LOD-490)	164 11.1-23400	<LOD (<LOD-107)	267 (64.1-4680)
DnOP	<LOD	-	<LOD	-
DiNP	<LOD (<LOD-1440)	-	<LOD (<LOD-452)	-
DiDP	<LOD	-	<LOD	-
∑PAEs	77.1 (<LOD-1936)	1830 (168-34500)	<LOD (<LOD-559)	1130 (621-6160)

Table S19 - Plastic additives emissions to the environment from menstrual products used by women that menstruate in Spain in kg/year.

Compound	Sanitary pads	Panty liners	Tampons	Reusable sanitary pads	Menstrual underwear	Menstrual cups
	Median (range)	Median (range)	Median (range)	Median (range)	Median (range)	Median (range)
BBzP	0.000 (0.000-10.40)	0.531 (0.000-3.906)	0.531 (0.000-3.906)	0.000	0.000	0.000
DBP	0.000	0.000 (0.000-30.17)	0.000	0.000 (0.000-0.031)	0.000 (0.000-0.132)	0.002 (0.000-0.005)
DEHP	0.377 (0.000-18.07)	0.567 (0.00-11.76)	1.072 (0.000-8.292)	5.398 (0.530-11.14)	0.067 (0.000-0.235)	0.002 (0.001-0.019)
DiNP	8.644 (0.000-8440)	6.896 (0.000-42.02)	3.442 (0.000-90.92)	0.000 (0.000-3.007))	0.000 (0.000-0.871)	0.000 (0.000-0.020)
DiDP	5.298 (0.210-176)	0.000 (0.000-1.936)	0.385 (0.000-6.833)	0.020 (0.012-0.052)	0.000	0.000 (0.000-0.001)
**∑PAEs**	**26.98 (2.627-11223)**	**10.66 (0.000-89.73)**	**7.670 (1.358-104.5)**	**6.643 (1.132-11.19)**	**0.118 (0.000-1.137)**	**0.013 (0.002-0.028)**
TEP	0.000 (0.000-1.495)	0.000 (0.000-2.200)	0.000 (0.000-0.657)	0.000 (0.000-0.001)	0.000	0.000 (0.000-0.001)
TCEP	0.000	0.000	844.6 (448.9-3848)	0.000	0.000 (0.000-127.4)	0.000
TPPO	0.000 (0.000-246.0)	218.1 (0.000-3898)	0.000	0.000	0.000	0.000
TCIPP	0.000 (0.000-1611)	0.000 (0.000-9796)	0.000	0.000	0.000 (0.000-132.2)	0.000
TDCIPP	0.000 (0.000-882.7)	1222 (0.000-13382)	0.000	0.000	0.000	0.000
TPHP	40.91 (0.000-715.1)	20.75 (0.000-267.5)	114.3 (5.959-454.5)	211.6 (29.92-470.7)	176.3 (3.597-555.3)	0.000
TNBP	7.827 (2.296-328.8)	1.373 (0.000-139.3)	5.743 (0.000-13.42)	0.000	0.000	0.000
DCP	0.000 (0.000-0.110)	0.059 (0.000-0.260)	0.000 (0.000-0.257)	0.000	0.000	0.000
TBOEP	0.000	0.000 (0.000-0.438)	0.000	0.000	0.000	0.000
RDP	0.000 (0.000-0.006)	0.000 (0.000-0.022)	0.000	0.000	0.000	0.000
2IPPDPP	0.000 (0.000-0.135)	0.000 (0.000-0.161)	0.000	0.000	0.000	0.000
4IPPDPP	0.000 (0.000-3.479)	0.001 (0.000-0.052)	0.000	0.000	0.000	0.000
TCP	0.000	0.001 (0.000-0.051)	0.000	0.000	0.000	0.000
EHDPP	0.000	0.076 (0.000-0.564)	0.000 (0.000-0.365)	0.086 (0.000-0.182)	0.000	0.000
B4IPPP	0.000	0.000 (0.000-0.046)	0.000	0.000	0.000	0.000
TEHP	0.000 (0.000-0.381)	0.000 (0.000-1.033)	0.000 (0.000-0.045)	0.158 (0.000-0.373)	0.000 (0.000-0.009)	0.000
**∑OPEs**	**10.28 (3.017-362.9)**	**4.221 (0.801-139.3)**	**7.365 (0.938-15.58)**	**0.501 (0.031-0.936)**	**0.311 (0.004-0.555)**	**0.000 (0.000-0.001)**
DIPA	0.000	0.671	0.265	0.000	0.000	0.000

	(0.000-0.169)	(0.000-2.117)	(0.000-0.983)			
TBC	0.580<br>(0.163-5.736)	0.121<br>(0.000-29.66)	25.62<br>(0.195-80.34)	0.000<br>(0.000-0.038)	0.000<br>(0.000-0.009)	0.000
DBA	0.000<br>(0.000-0.010)	1.361<br>(0.000-6.077)	1.393<br>(0.000-6.588)	0.000	0.000	0.000<br>(0.000-0.005)
ATBC	169.3<br>(48.20-52938)	8.994<br>(0.115-1936)	291.2<br>(6.468-1437)	0.000<br>(0.000-0.024)	0.000	0.001<br>(0.000-0.002)
DEHA	2.312<br>(0.000-2876)	0.000<br>(0.000-24.79)	1.371<br>(0.233-20.74)	0.195<br>(0.016-0.455)	0.113<br>(0.062-1.134)	0.001<br>(0.000-0.002)
DINA	0.000<br>(0.000-0.150)	0.000<br>(0.000-3.354)	0.000<br>(0.000-0.260)	0.000	0.000	0.000
DINCH	0.000<br>(0.000-4.187)	0.000<br>(0.000-36.82)	0.852<br>(0.000-355.5)	0.000	0.000	0.000<br>(0.000-0.003)
**∑APs**	**195.1<br>(50.36-83407)**	**13.88<br>(1.308-2027)**	**366.1<br>(9.609-1545)**	**0.207<br>(0.065-0.455)**	**0.117<br>(0.062-1.134)**	**0.003<br>(0.000-0.005)**
**∑TOTAL**	**225.2<br>(76.87-213127)**	**82.06<br>(4.959-2039)**	**471.5<br>(12.07-1560)**	**7.334<br>(1.372-12.47)**	**1.055<br>(0.119-1.754)**	**0.015<br>(0.004-0.033)**

Table S20 – PAEs concentrations (ng/product) in sanitary pads, panty liners, tampons, reusable sanitary pads, menstrual underwear and menstrual cups.

	**Sanitary pads (n= 10)**		**Panty liners (n= 8)**		**Tampons (n= 9)**		**Reusable sanitary pads (n= 4)**		**Menstrual underwear (n= 4)**		**Menstrual cups (n= 6)**	
**Compound**	DF (%)	Median; Mean<br>Range	DF (%)	Median; Mean<br>Range	DF (%)	Median; Mean<br>Range	DF (%)	Median; Mean<br>Range	DF (%)	Median; Mean<br>Range	DF (%)	Median; Mean<br>Range
MEHP	0	<LOD; <LOD<br><LOD	0	<LOD; <LOD<br><LOD	0	<LOD; <LOD<br><LOD	0	<LOD; <LOD<br><LOD	0	<LOD; <LOD<br><LOD	0	<LOD; <LOD<br><LOD
BBzP	0	<LOD; <LOD<br><LOD	25	<LOD; 5.82<br><LOD-29.7	11	<LOD; <LOD<br><LOD-8.86	0	<LOD<br><LOD	0	<LOD; <LOD<br><LOD	0	<LOD<br><LOD
DBP	0	<LOD; <LOD<br><LOD	37	<LOD; 335<br><LOD-1700	0	<LOD; <LOD<br><LOD	25	<LOD; 956<br><LOD;3164	25	<LOD; 3752<br><LOD-13564	83	2005; 2106<br><LOD-4235
DHexP	0	<LOD; <LOD<br><LOD	0	<LOD; <LOD<br><LOD	0	<LOD; <LOD<br><LOD	0	<LOD; <LOD<br><LOD	0	<LOD; <LOD<br><LOD	0	<LOD; <LOD<br><LOD
DEHP	10	<LOD; 501<br><LOD-4887	37	<LOD; 229<br><LOD-662	11	<LOD; 31.7<br><LOD-232	100	555139; 577495<br>54534-1145166	50	6935; 9531<br><LOD-24192	100	1859; 3848<br>433-15444
DnOP	0	<LOD; <LOD<br><LOD	0	<LOD; <LOD<br><LOD	0	<LOD; <LOD<br><LOD	0	<LOD; <LOD<br><LOD	0	<LOD; <LOD<br><LOD	0	<LOD; <LOD<br><LOD
DiNP	40	<LOD; 2239<br><LOD-8776	25	<LOD; 420<br><LOD-2095	22	<LOD; 280<br><LOD-1163	25	<LOD; 78081<br><LOD-309252	25	<LOD; 24024<br><LOD-89540	33	<LOD, 5241<br><LOD-16713
DiDP	40	<LOD; 3291<br><LOD-20784	0	<LOD; <LOD<br><LOD	0	<LOD; <LOD<br><LOD	100	2076; 2708<br>1282-5398	0	<LOD; <LOD<br><LOD	50	<LOD; <LOD<br><LOD-769
**∑PAEs**	**60**	**3759; 6060<br><LOD-26441**	**50**	**584; 1094<br><LOD-2896**	**33**	**<LOD; 445<br><LOD-1332**	**100**	**683745; 659280<br>117621-1152010**	**50**	**16120; 38258<br><LOD-117697**	**100**	**10825; 11615<br>1676-22978**

DF=detection frequency

Table S21 – OPEs concentrations (ng/product) sanitary pads, panty liners, tampons, reusable sanitary pads, menstrual underwear and menstrual cups.

Compound	Sanitary pads<br>(n= 10)		Panty liners<br>(n= 8)		Tampons<br>(n= 9)		Reusable sanitary pads<br>(n= 4)		Menstrual underwear<br>(n= 4)		Menstrual cups<br>(n= 6)	
	DF<br>(%)	Median; Mean<br>Range	DF<br>(%)	Median; Mean<br>Range	DF<br>(%)	Median; Mean<br>Range	DF<br>(%)	Median; Mean<br>Range	DF<br>(%)	Median; Mean<br>Range	DF<br>(%)	Median; Mean<br>Range
TEP	20	<LOD; 71.7<br><LOD-407	25	<LOD; 25.0<br><LOD-124	33	<LOD; 12.9<br><LOD-37.0	0	<LOD; <LOD<br><LOD	0	<LOD; <LOD<br><LOD	17	<LOD; 197<br><LOD-1067
TCEP	0	<LOD; <LOD<br><LOD	0	<LOD; <LOD<br><LOD	100	47.6; 71.6<br>25.3-217	0	<LOD; <LOD<br><LOD	25	<LOD; 3335<br><LOD-13104	0	<LOD; <LOD<br><LOD
TPPO	10	<LOD; 6.88<br><LOD-67.1	50	12.3; 50.0<br><LOD-220	0	<LOD; <LOD<br><LOD	0	<LOD; <LOD<br><LOD	0	<LOD; <LOD<br><LOD	0	<LOD; <LOD<br><LOD
TCIPP	0	<LOD; <LOD<br><LOD	25	<LOD; 99.4<br><LOD-552	0	<LOD; <LOD<br><LOD	0	<LOD; <LOD<br><LOD	25	<LOD; 3414<br><LOD-13594	0	<LOD; <LOD<br><LOD
TPrP	0	<LOD; <LOD<br><LOD	0	<LOD; <LOD<br><LOD	0	<LOD; <LOD<br><LOD	0	<LOD; <LOD<br><LOD	0	<LOD; <LOD<br><LOD	0	<LOD; <LOD<br><LOD
TDCIPP	10	<LOD; 26.6<br><LOD-241	50	69.5; 184<br><LOD; 754	0	<LOD; <LOD<br><LOD	0	<LOD; <LOD<br><LOD	0	<LOD; <LOD<br><LOD	0	<LOD; <LOD<br><LOD
TPHP	30	<LOD; 10.3<br><LOD-54.0	37	<LOD; 4.34<br><LOD-15.1	33	<LOD, 0.58<br><LOD-1.53	100	21756; 23749<br>3077-48408	100	18128, 23434<br>370-57110	0	<LOD; <LOD<br><LOD
TNBP	100	869; 971<br>469-1660	75	58.6; 83.2<br><LOD-261	78	39.3; 53.1<br><LOD-262	0	<LOD; <LOD<br><LOD	0	<LOD; <LOD<br><LOD	0	<LOD; <LOD<br><LOD
DCP	30	<LOD; 8.40<br><LOD-28.4	50	3.78; 5.45<br><LOD-14.7	0	<LOD; <LOD<br><LOD	0	<LOD; <LOD<br><LOD	0	<LOD; <LOD<br><LOD	0	<LOD; <LOD<br><LOD
TBOEP	0	<LOD; <LOD<br><LOD	12	<LOD; 3.47<br><LOD-24.7	0	<LOD; <LOD<br><LOD	0	<LOD; <LOD<br><LOD	0	<LOD; <LOD<br><LOD	0	<LOD; <LOD<br><LOD
RDP	10	<LOD; 0.33<br><LOD-1.61	12	<LOD; 0.23<br><LOD-1.24	0	<LOD; <LOD<br><LOD	0	<LOD; <LOD<br><LOD	0	<LOD; <LOD<br><LOD	0	<LOD; <LOD<br><LOD
2IPPDPP	10	<LOD; 3.89<br><LOD-36.7	25	<LOD; 2.12<br><LOD-9.08	0	<LOD; <LOD<br><LOD	0	<LOD; <LOD<br><LOD	0	<LOD; <LOD<br><LOD	0	<LOD; <LOD<br><LOD
4IPPDPP	10	<LOD; 95.2<br><LOD-948	25	<LOD; 0.83<br><LOD-2.95	0	<LOD; <LOD<br><LOD	0	<LOD; <LOD<br><LOD	0	<LOD; <LOD<br><LOD	0	<LOD; <LOD<br><LOD
TCP	0	<LOD; <LOD<br><LOD	25	<LOD; 0.82<br><LOD-2.91	0	<LOD; <LOD<br><LOD	0	<LOD; <LOD<br><LOD	0	<LOD; <LOD<br><LOD	0	<LOD; <LOD<br><LOD
EHDPP	0	<LOD; <LOD<br><LOD	50	5.06; 9.67<br><LOD-31.8	11	<LOD; 3.78<br><LOD-20.6	75	8893; 9121<br><LOD-18681	0	<LOD; <LOD<br><LOD	0	<LOD; <LOD<br><LOD
B4IPPP	0	<LOD; <LOD<br><LOD	12	<LOD; 0.63<br><LOD-2.57	0	<LOD; <LOD<br><LOD	0	<LOD; <LOD<br><LOD	0	<LOD; <LOD<br><LOD	0	<LOD; <LOD<br><LOD
TEHP	40	<LOD; 42.4<br><LOD-103	25	<LOD; 24.4<br><LOD-58.2	0	<LOD; <LOD<br><LOD	75	16281; 17764<br><LOD-38351	25	<LOD; 477<br><LOD-954	0	<LOD; <LOD<br><LOD
**∑OPEs**	**100**	**1369; 1268<br>549-2043**	**100**	**214; 499<br>77.2-1853**	**100**	**118; 167<br>71.3-548**	**100**	**51748; 50872<br>3451-96541**	**100**	**32706; 31095<br>1122-57846**	**17**	**<LOD; 319<br><LOD-1177**

Table S22 – APs concentrations (ng/product) in sanitary pads, panty liners, tampons, reusable sanitary pads, menstrual underwear and menstrual cups.

	**Sanitary pads (n= 10)**		**Panty liners (n= 8)**		**Tampons (n= 9)**		**Reusable sanitary pads (n= 4)**		**Menstrual underwear (n= 4)**		**Menstrual cups (n= 6)**	
**Compound**	DF (%)	Median; Mean<br>Range	DF (%)	Median; Mean<br>Range	DF (%)	Median; Mean<br>Range	DF (%)	Median; Mean<br>Range	DF (%)	Median; Mean<br>Range	DF (%)	Median; Mean<br>Range
ATEC	0	<LOD; <LOD<br><LOD	0	<LOD; <LOD<br><LOD	0	<LOD; <LOD<br><LOD	0	<LOD; <LOD<br><LOD	0	<LOD; <LOD<br><LOD	0	<LOD; <LOD<br><LOD
DIPA	10	<LOD; 4.60<br><LOD-46.0	87	33.2; 37.5<br><LOD-119	33	<LOD; 3.17<br><LOD-11.2	0	<LOD; <LOD<br><LOD	0	<LOD; <LOD<br><LOD	0	<LOD; <LOD<br><LOD
TBC	100	81.2; 90.6<br>25.2-177	62	6.04; 96.5<br><LOD-508	44	<LOD; 1.91<br><LOD-7.07	25	<LOD; 1241<br><LOD-4963	25	<LOD; 241<br><LOD-965	0	<LOD; <LOD<br><LOD
DBA	0	<LOD; <LOD<br><LOD	37	<LOD; 21.9<br><LOD-116	0	<LOD; <LOD<br><LOD	0	<LOD; <LOD<br><LOD	0	<LOD; <LOD<br><LOD	17	<LOD; 738<br><LOD-4430
ATBC	100	31529; 31660<br>11210-50565	75	473; 7206<br><LOD-24423	78	39.6; 153<br><LOD-539	25	<LOD; 620<br><LOD-2482	0	<LOD; <LOD<br><LOD	100	600; 686<br>67.3-1739
DEHA	30	<LOD; 5402<br><LOD-38834	0	<LOD; <LOD<br><LOD	0	<LOD; <LOD<br><LOD	100	20081; 22148<br>1672-46760	100	11574; 36546<br>6402-116632	50	452; 599<br><LOD-1705
BTHC	0	<LOD; <LOD<br><LOD	0	<LOD; <LOD<br><LOD	0	<LOD; <LOD<br><LOD	0	<LOD; <LOD<br><LOD	0	<LOD; <LOD<br><LOD	0	<LOD; <LOD<br><LOD
DINA	10	<LOD, 4.10<br><LOD-41.0	37	<LOD; 26.4<br><LOD-188	0	<LOD; <LOD<br><LOD	0	<LOD; <LOD<br><LOD	0	<LOD; <LOD<br><LOD	0	<LOD; <LOD<br><LOD
DINCH	0	<LOD; <LOD<br><LOD	37	<LOD; 310<br><LOD-2057	22	<LOD; 109<br><LOD-759	0	<LOD; <LOD<br><LOD	0	<LOD; <LOD<br><LOD	33	<LOD; 527<br><LOD-2585
**∑APs**	**100**	**39324; 37161<br>11235-60691**	**100**	**741; 7699<br>58.4-24980**	**100**	**73.0; 267<br>6.93-1249**	**100**	**21322; 24010<br>6634-46760**	**100**	**12057; 36787<br>6402-116632**	**100**	**2577; 2550<br>256-4498**

DF=detection frequency

References

[1] J. Fernández-Arribas, S. Callejas-Martos, A. Balasch, T. Moreno, and E. Eljarrat, "Simultaneous analysis of several plasticizer classes in different matrices by on-line turbulent flow chromatography-LC–MS/MS," *Anal Bioanal Chem*, Oct. 2024, doi: 10.1007/s00216-024-05593-2.

[2] C. Parent *et al.*, "Menstrual hygiene products: A practice evaluation," *J Gynecol Obstet Hum Reprod*, vol. 51, no. 1, Jan. 2022, doi: 10.1016/j.jogoh.2021.102261.

[3] S. Fourcassier, M. Douziech, P. Pérez-López, and L. Schiebinger, "Menstrual products: A comparable Life Cycle Assessment," *Cleaner Environmental Systems*, vol. 7, Dec. 2022, doi: 10.1016/j.cesys.2022.100096.

[4] A. I. L. García, C. Moráis-Moreno, M. de L. Samaniego-Vaesken, A. M. Puga, G. Varela-Moreiras, and T. Partearroyo, "Association between hydration status and body composition in healthy adolescents from Spain," *Nutrients*, vol. 11, no. 11, Nov. 2019, doi: 10.3390/nu11112692.

[5] A. M. López-Sobaler *et al.*, "Overweight and general and abdominal obesity in a representative sample of Spanish adults: Findings from the ANIBES Study," *Biomed Res Int*, vol. 2016, 2016, doi: 10.1155/2016/8341487.

[6] "US EPA. Integrated Risk Information System (IRIS). https://www.epa.gov/iris (accessed: 19.02.2025)."

[7] "US EPA. Provisional Peer-Reviewed Toxicity Values (PPRTVs). https://www.epa.gov/pprtv (accessed: 19.02.2025)."

[8] "ATSDR. Minimal Risk Levels (MRLs) for Hazardous Substances. https://wwwn.cdc.gov/TSP/MRLS/mrlsListing.aspx (accessed: 19.02.2025)."

[9] V. Silano *et al.*, "Update of the risk assessment of di-butylphthalate (DBP), butyl-benzyl-phthalate (BBP), bis(2-ethylhexyl)phthalate (DEHP), di-isononylphthalate (DINP) and di-isodecylphthalate (DIDP) for use in food contact materials," *EFSA Journal*, vol. 17, no. 12, Dec. 2019, doi: 10.2903/j.efsa.2019.5838.

[10] N. Van den Eede, A. C. Dirtu, H. Neels, and A. Covaci, "Analytical developments and preliminary assessment of human exposure to organophosphate flame retardants from indoor dust," *Environ Int*, vol. 37, no. 2, pp. 454–461, 2011, doi: 10.1016/j.envint.2010.11.010.

[11] C. He *et al.*, "Organophosphate and brominated flame retardants in Australian indoor environments: Levels, sources, and preliminary assessment of human exposure," *Environmental Pollution*, vol. 235, pp. 670–679, Apr. 2018, doi: 10.1016/j.envpol.2017.12.017.

[12] "Opinion of the Scientific Panel on food additives, flavourings, processing aids and materials in contact with food (AFC) related to the 12th list of substances for food contact materials," *EFSA Journal*, vol. 4, no. 10, Oct. 2006, doi: 10.2903/j.efsa.2006.395.

[13] "Opinion of the Scientific Panel on food additives, flavourings, processing aids and materials in contact with food (AFC) on the application of a Total Reduction Factor of 5 for di(2-

ethylhexyl)adipate used as plasticiser in flexible PVC food packaging films," *EFSA Journal*, vol. 3, no. 9, Sep. 2005, doi: 10.2903/j.efsa.2005.217.

[14] C. J. Gao and K. Kannan, "Phthalates, bisphenols, parabens, and triclocarban in feminine hygiene products from the United States and their implications for human exposure," *Environ Int*, vol. 136, Mar. 2020, doi: 10.1016/j.envint.2020.105465.

[15] Z. Tang, M. Chai, J. Cheng, Y. Wang, and Q. Huang, "Occurrence and Distribution of Phthalates in Sanitary Napkins from Six Countries: Implications for Women's Health," *Environ Sci Technol*, vol. 53, no. 23, pp. 13919–13928, Dec. 2019, doi: 10.1021/acs.est.9b03838.

[16] C. J. Gao, F. Wang, H. M. Shen, K. Kannan, and Y. Guo, "Feminine Hygiene Products - A Neglected Source of Phthalate Exposure in Women," *Environ Sci Technol*, vol. 54, no. 2, pp. 930–937, Jan. 2020, doi: 10.1021/acs.est.9b03927.
